# Phylogenetic and functional analyses of *N*^6^-methyladenosine RNA methylation factors in the wheat scab fungus *Fusarium graminearum*

**DOI:** 10.1128/msphere.00552-23

**Published:** 2023-12-12

**Authors:** Hyeonjae Kim, Jianzhong Hu, Hunseung Kang, Wonyong Kim

**Affiliations:** 1 Korean Lichen Research Institute, Sunchon National University, Suncheon, South Korea; 2 Department of Applied Biology, College of Agriculture and Life Sciences, Chonnam National University, Gwangju, South Korea; University College Dublin, Dublin, Ireland

**Keywords:** *N*^6^-methyladenosine, m^6^A RNA methylation, MT-A70, *MTA1*, *Fusarium graminearum*

## Abstract

In eukaryotes, *N*^6^-methyladenosine (m^6^A) RNA modification plays a crucial role in governing the fate of RNA molecules and has been linked to various developmental processes. However, the phyletic distribution and functions of genetic factors responsible for m^6^A modification remain largely unexplored in fungi. To get insights into the evolution of m^6^A machineries, we reconstructed global phylogenies of potential m^6^A writers, readers, and erasers in fungi. Substantial copy number variations were observed, ranging from up to five m^6^A writers in early-diverging fungi to a single copy in the subphylum Pezizomycotina, which primarily comprises filamentous fungi. To characterize m^6^A factors in a phytopathogenic fungus *Fusarium graminearum*, we generated knockout mutants lacking potential m^6^A factors including the sole m^6^A writer *MTA1*. However, the resulting knockouts did not exhibit any noticeable phenotypic changes during vegetative and sexual growth stages. As obtaining a homozygous knockout lacking *MTA1* was likely hindered by its essential role, we generated *MTA1*-overexpressing strains (*MTA1*-OE). The *MTA1*-OE5 strain showed delayed conidial germination and reduced hyphal branching, suggesting its involvement during vegetative growth. Consistent with these findings, the expression levels of *MTA1* and a potential m^6^A reader *YTH1* were dramatically induced in germinating conidia, followed by the expression of potential m^6^A erasers at later vegetative stages. Several genes including transcription factors, transporters, and various enzymes were found to be significantly upregulated and downregulated in the *MTA1*-OE5 strain. Overall, our study highlights the functional importance of the m^6^A methylation during conidial germination in *F. graminearum* and provides a foundation for future investigations into m^6^A modification sites in filamentous fungi.

**IMPORTANCE:**

*N*^6^-methyladenosine (m^6^A) RNA methylation is a reversible posttranscriptional modification that regulates RNA function and plays a crucial role in diverse developmental processes. This study addresses the knowledge gap regarding phyletic distribution and functions of m^6^A factors in fungi. The identification of copy number variations among fungal groups enriches our knowledge regarding the evolution of m^6^A machinery in fungi. Functional characterization of m^6^A factors in a phytopathogenic filamentous fungus *Fusarium graminearum* provides insights into the essential role of the m^6^A writer *MTA1* in conidial germination and hyphal branching. The observed effects of overexpressing *MTA1* on fungal growth and gene expression patterns of m^6^A factors throughout the life cycle of *F. graminearum* further underscore the importance of m^6^A modification in conidial germination. Overall, this study significantly advances our understanding of m^6^A modification in fungi, paving the way for future research into its roles in filamentous growth and potential applications in disease control.

## INTRODUCTION

Eukaryotic RNA undergoes over 100 chemical modifications that can impact RNA processing and metabolism (1–3). These modifications include mRNA capping, mRNA polyadenylation, RNA splicing, and RNA methylation (4). Among various RNA methylation, *N*^6^-methyladenosine (m^6^A) is characterized by the methylation of the sixth nitrogen atom on adenosine within RNA. m^6^A was first described in mammalian cells 50 years ago and is the most prevalent and abundant modification found in eukaryotic mRNA (5, 6). This modification is reversible and regulated by a set of enzymes that function as writers (methyltransferases), erasers (demethylases), and readers (RNA-binding proteins that recognize m^6^A) (7). Due to its reversible nature, m^6^A modification serves as a rapid response to environmental stress and regulates various processes including development, immune reactions, and cancer progression in animals, fungi and plants (8–15).

m^6^A RNA modification is estimated to occur in approximately 0.1%–0.4% of adenosine nucleotides found in mammalian mRNAs (16, 17). In mammals, the methyltransferase complex responsible for m^6^A methylation includes proteins, such as methyltransferase-like protein 3 (METTL3), methyltransferase-like protein 14 (METTL 14), and the pre-mRNA-splicing regulator Wilms tumor 1-associated protein (WTAP) (18–20). METTL3 serves as the catalytic subunit, forming a heterodimer with its paralogue, METTL14. WTAP is a regulatory subunit whose function is to recruit the m^6^A methyltransferase complex to the target mRNA in nuclear speckles and is believed to act as a bridge between the METTL3/METTL14 heterodimer and accessory proteins (21). Among the accessory proteins, virilizer-like methyltransferase-associated protein (VIRMA) is implicated in stabilizing the methyltransferase complex and plays a role in the selection of specific sites for the m^6^A modification (22). YT521-B homology (YTH)-domain proteins are known as m^6^A readers located in the cytoplasm that influence on translation of methylated mRNAs and their subsequent degradation (23, 24). In humans, the YTH domain family 2 protein (YTHDF2) is involved in controlling mRNA stability by specifically binding to m^6^A-modified mRNAs and relocating them from ribosomes to processing bodies (25). Demethylation of m^6^A is catalyzed by 2-oxoglutarate and iron-dependent [2OG-Fe(II)] dioxygenases AlkB-like domain-containing proteins and the fat mass and obesity-associated protein in mammals (26, 27). These diverse m^6^A factors collectively contribute to m^6^A RNA modification, playing a significant role in RNA metabolism and gene regulation.

In the budding yeast *Saccharomyces cerevisiae*, the m^6^A writer IME4 (Inducer of meiosis 4) is homologous to human METTL3 that catalyzes m^6^A modification of specific target RNAs involved in the initiation of sexual development and sporulation (28). IME4 is required for proper entry into sexual development and progression through meiotic divisions in diploid cells and is also known to regulate triacylglycerol metabolism, vacuolar morphology, and mitochondrial morphology in haploid cells (29, 30). A yeast two-hybrid screening identified core components of the methyltransferase complex composed of two catalytic factors IME4 and KAR4 (Karyogamy 4, orthologous to human METTL14), as well as two non-catalytic factors MUM2 (Muddled meiosis 2, orthologous to human WTAP), and SLZ1 (Sporulation leucine zipper 1 orthologous to human ZCH13H3) in the budding yeast (31). Recent work revealed that an interacting protein with KAR4, Ygl036wp (thereafter, coined VIR1), in the budding yeast shares a folding pattern similar to VIRMA, despite the lack of discernable protein domains (32, 33). In the budding yeast, SLZ1 enables the methyltransferase complex comprising IME4, KAR4, MUM2 (WTAP), and VIR1 to function in m^6^A deposition (33).

The genome of the budding yeast encodes a single YTH domain-containing protein, PHO92 (Phosphate metabolism 92), that has been originally described to be involved in phosphate metabolism and response (34, 35). Recently, it became clear that PHO92 recognizes m^6^A-modified transcripts, facilitates protein synthesis and subsequent decay of m^6^A-modified transcripts, and promotes meiotic recombination (36, 37). In the fission yeast *Schizosaccharomyces pombe*, a YTH domain-containing protein, Mmi1 (Meiotic mRNA interception 1), was involved in the selective elimination of meiosis-specific transcripts *via* posttranscriptional gene silencing (38). However, Mmi1 cannot bind to the m^6^A consensus motif, suggesting that the function of YTH domain-containing proteins is not limited to m^6^A recognition and is implicated in diverse cellular functions (39). Although m^6^A writers and m^6^A readers have been studied in yeasts, to our best knowledge, m^6^A erasers await their discovery in fungi.

*Fusarium graminearum* is a filamentous phytopathogenic fungus and causes devastating diseases in our staple crops, such as wheat, barley, and corn (40). The fungus has served as an excellent model organism for investigating various biological aspects, including host-pathogen interactions, sexual development, mycotoxin production, and RNA editing (41–45). Recently, in the rice blast fungus *Magnaporthe oryzae*, MT-A70 domain protein 1 (MTA1, orthologous to human METTL4) was shown to play an important role in appressorium formation during infection process *via* regulation of autophagy process (46, 47). However, the roles of the m^6^A factors are poorly understood in *F. graminearum* and other filamentous fungi. Thus, the aims of this study were to (i) investigate phyletic distribution and copy number variations of m^6^A factors in the kingdom Fungi, (ii) functionally characterize putative m^6^A factors in *F. graminearum*, and (iii) identify potential targets of the m^6^A writer MTA1, which is the sole m^6^A writer found in the Pezizomycotina.

## RESULTS

### Divergence of m^6^A writers in the kingdom Fungi

**Fig 1:**
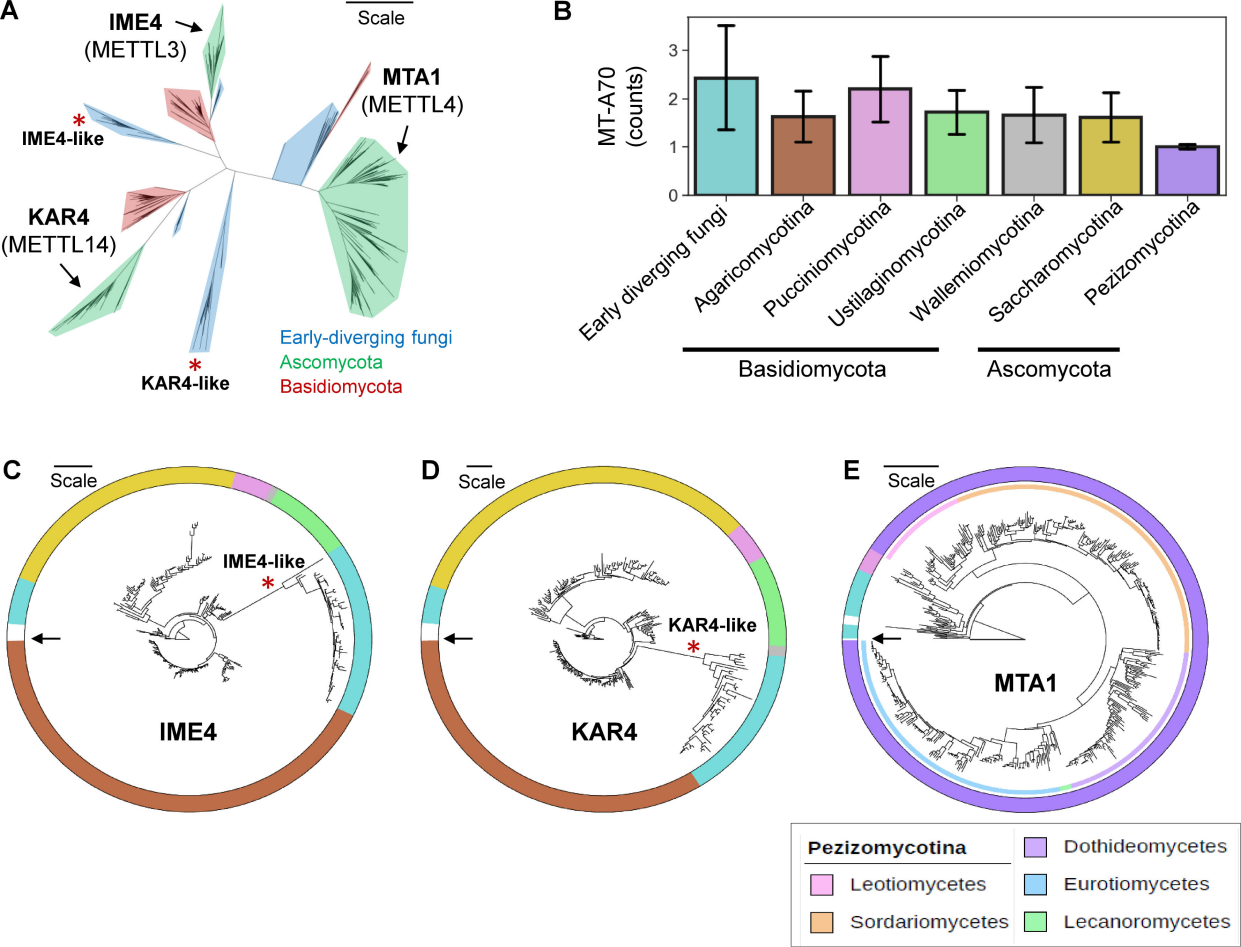
Phylogeny of potential m^6^A writers in fungi. (**A**) The maximum-likelihood phylogeny was estimated from 1,116 sequences of MT-A70 domain-containing proteins identified in 829 fungal species. Different phyla were shaded in blue for early-diverging fungi, red for Basidiomycota, and green for Ascomycota. Arrows indicate clades including previously characterized IME4, KAR4, and MTA1 in Ascomycota. Branches marked with red asterisks suggest clades that likely diverged at earlier time points (IME4-like and KAR4-like). The branch lengths in the tree reflect the amount of evolutionary change, with the scale indicating 1.0 amino acid sequence substitution per site. (**B**) Phyletic distribution of MT-A70 domain-containing proteins in fungi. The bars indicate the standard deviation of gene counts. (**C**) The phylogeny was estimated from 308 protein sequences that belong to the IME4 subclade and from five METTL3 orthologs found in metazoans and a model plant *Arabidopsis thaliana*. (**D**) The phylogeny was estimated from 298 protein sequences that belong to the KAR4 subclade and from five METTL14 orthologs found in metazoans and a model plant *Arabidopsis thaliana*. (**E**) The phylogeny was estimated from 510 protein sequences that belong to the MTA1 subclade and from five METTL4 orthologs found in metazoans and a model plant *Arabidopsis thaliana*. The inner color strip represents the fungal class within the subphylum Pezizomycotina, with each color indicating a distinct taxonomic group as shown in the inset box. (C–E) The color strip outside the tree represents different fungal taxa, with each color indicating a distinct taxonomic group as shown in Fig. 1B. The arrow indicates METTL homologs found in *Arabidopsis thaliana*, and the scale indicates 1.0 amino acid sequence substitution per site. More detailed trees showing UniProt protein IDs and bootstrap values are shown in Fig. S1.

To resolve phylogenetic relationships of genes encoding an MT-A70 protein domain (Pfam domain: PF05063), we collected 1,568 protein sequences from the UniProt database (accessed on 21 May 2023; https://www.uniprot.org/) and curated the list of MT-A70-containing proteins, excluding redundant entries or sequences with an *E*-value of smaller than 10^−5^ for PF05063 (see Section Materials and Methods). Following the manual curation, a maximum likelihood tree was reconstructed for 1,116 protein sequences containing an MT-A70 domain in 829 diverse fungal species. This tree revealed the presence of three distinct clades, each including the three previously characterized m^6^A writers in fungi: IME4, KAR4, and MTA1 (Fig. 1A). Within the IME4 and KAR4 clades, there was a branch exhibiting an early divergence, which exclusively consists of sequences derived from early-diverging fungi, such as species belonging to the phyla Chytridiomycota, Mucoromycota, and Zoopagomycota (see clades labeled with red asterisks in Fig. 1A). Notably, distinct patterns were observed in the phyletic distribution of IME4, KAR4, and MTA1 among different taxonomic groups in fungi (Fig. 1B). Some early-diverging fungi and species within the Pucciniomycotina subphylum were found to possess all the three MT-A70-containing proteins, IME4, KAR4, and MTA1, although many are lacking one of these proteins. The Basidiomycota species (mushroom-forming fungi) and Saccharomycotina species (budding yeasts) typically had IME4 and KAR4, whereas nearly all of the Pezizomycotina species that are comprised of entirely filamentous fungi were found to possess MTA1 as a sole MT-A70-containing protein.

Independently reconstructed maximum likelihood trees were generated for the IME4, KAR4, and MTA1 clades including their respective homologs of METTL3, METTL14, and METTL4, which were found in humans (*Homo sapiens*), mouse (*Mus musculus*), zebrafish (*Danio rerio*), fruit fly (*Drosophila melanogaster*), and a model plant (*Arabidopsis thaliana*) serving as an outgroup (Fig. 1C through E; see Fig. S1 for bootstrap values and protein IDs). The phylogenetic patterns of the IME4 and KAR4 clades exhibited resemblance, encompassing early-diverging fungi and species from Basidiomycota and Saccharomycotina. Among species possessing either IME4 (294 species) or KAR4 (286 species), three-quarters of the species (221 species) had both m^6^A writers (Table S1). As previously mentioned, some early-diverging fungi exhibited the presence of two copies of IME4 and KAR4. One of these copies appears to have undergone divergence before the emergence of Dikarya (commonly known as “higher fungi”), forming distinct clades here dubbed IME4-like and KAR4-like with robust 100% bootstrap support (Fig. S1). The phylogeny of MTA1 exhibited monophyly and was largely congruent to the phylogeny of Pezizomycotina (Fig. 1E).

**Fig 2:**
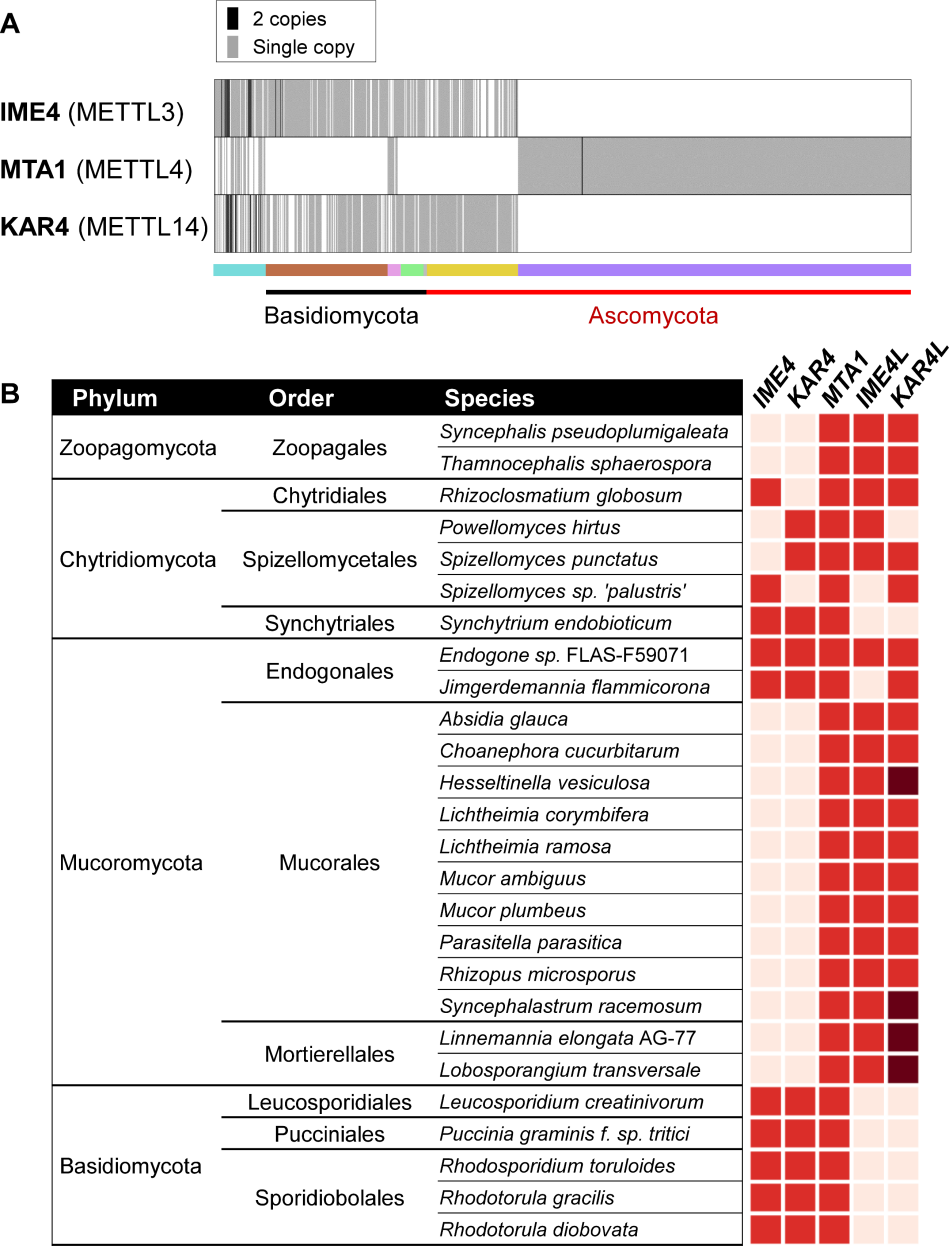
Phyletic distribution of m^6^A writers in fungi. (**A**) Copy number variation of potential m^6^A writers in fungi. The color strip represents different fungal taxa, with each color indicating a distinct taxonomic group as shown in Fig. 1B. (**B**) Phyletic distribution of IME4, KAR4, and MTA1 in early-diverging fungal phyla. Heatmaps showing the presence (red for one copy, and dark red for two copies) and absence (white) of genes encoding the MT-A70 domain. *IME4L: IME4-like* gene, and *KAR4L: KAR4-like* gene.

Species belonging to Pezizomycotina possessed MTA1 as a sole MT-A70-containing protein. On the other hand, many early-diverging fungi and species from Pucciniomycotina exhibited the presence of three m^6^A writers, IME4, KAR4, and MTA1 (Fig. 2A). We took a closer look at 26 species that featured the three m^6^A writers. The increased abundance of MT-A70 domain-containing proteins in early-diverging fungi can be attributable to the existence of *IME4-like* and *KAR4-like* genes that diverged from *MTA1* at earlier points in time. Although the distribution patterns of MT-A70 domain-containing proteins in early-diverging fungi that belong to the class Chytridiomycetes and Zoopagomycetes are highly variable, they all contained *IME4* (or *IME4-like*), *KAR4* (or *KAR4-like*), and *MTA1* (Fig. 2B). Species that belong to the Mucoromycota (except for species from the Endogonales) all lacked *IME4* and *KAR4*, instead having *IME4-like* and *KAR4-like*, suggesting that some lineages in the Mucorales have lost *IME4* and *KAR4* that can be found in species in the Glomeromycetes (the fungal Class sister to the Mucoromycetes). Interestingly, some species in the Mucorales and Mortierellales have two copies of *KAR4-like*, suggesting gene duplication events. Species that belong to the Pucciniomycotina, the earliest-diverging subphylum within Basidiomycota, have *IME4*, *KAR4*, and *MTA1* but are devoid of *IME4-like* and *KAR4-like* genes (Fig. 2B), suggesting that these early-diverged MT-A70 domain-containing genes were completely lost in Dikarya.

Recent studies revealed the components of yeast m^6^A methyltransferase complexes were highly conserved with those found in mammals, insects, and plants, including IME4, KAR4, MUM2 (a homolog of WTAP), and VIR1 (32, 33). Thus, we conducted a search in the UniProt database for relevant Pfam domains, specifically PF17098 for the WTAP/Mum2p family and PF15912 for the N-terminal domain of *virilizer* (hereafter coined VIRN). We identified 172 and 53 fungal proteins that harbor PF17098 for WTAP and PF15912 for VIRN, respectively, in the current UniProt database (accessed on 4 June 2023; Table S1). After manual curation, we reconstructed phylogenetic trees for WTAP and VIRN, together with homologs found in humans, mice, zebrafish, fruit flies, and *Arabidopsis thaliana* as an outgroup. WTAP orthologs including Mum2 in yeasts were found only in early-diverging fungi and species from Basidiomycota, and Saccharomycotina, lacking in Pezizomycotina species (Fig. S1). Only 37 fungal species that belong to early-diverging fungi and Basidiomycota were shown to have genes encoding a VIRN domain (Fig. S1). The paucity of homologous sequences in fungi may be attributable to the fact that WTAP and VIRN sequences have diverged significantly from the known sequences, making their identification challenging, especially in Ascomycetous fungi, such as Saccharomycotina and Pezizomycotina. Indeed, a homolog of the virilizer protein, VIR1, was recently found in the budding yeast, which lacks the apparent Pfam domain for VIRN but it exhibits a 3D structure similar to the human homolog VIRMA (32).

### Identification of putative m^6^A reader and eraser in fungi

**Fig 3:**
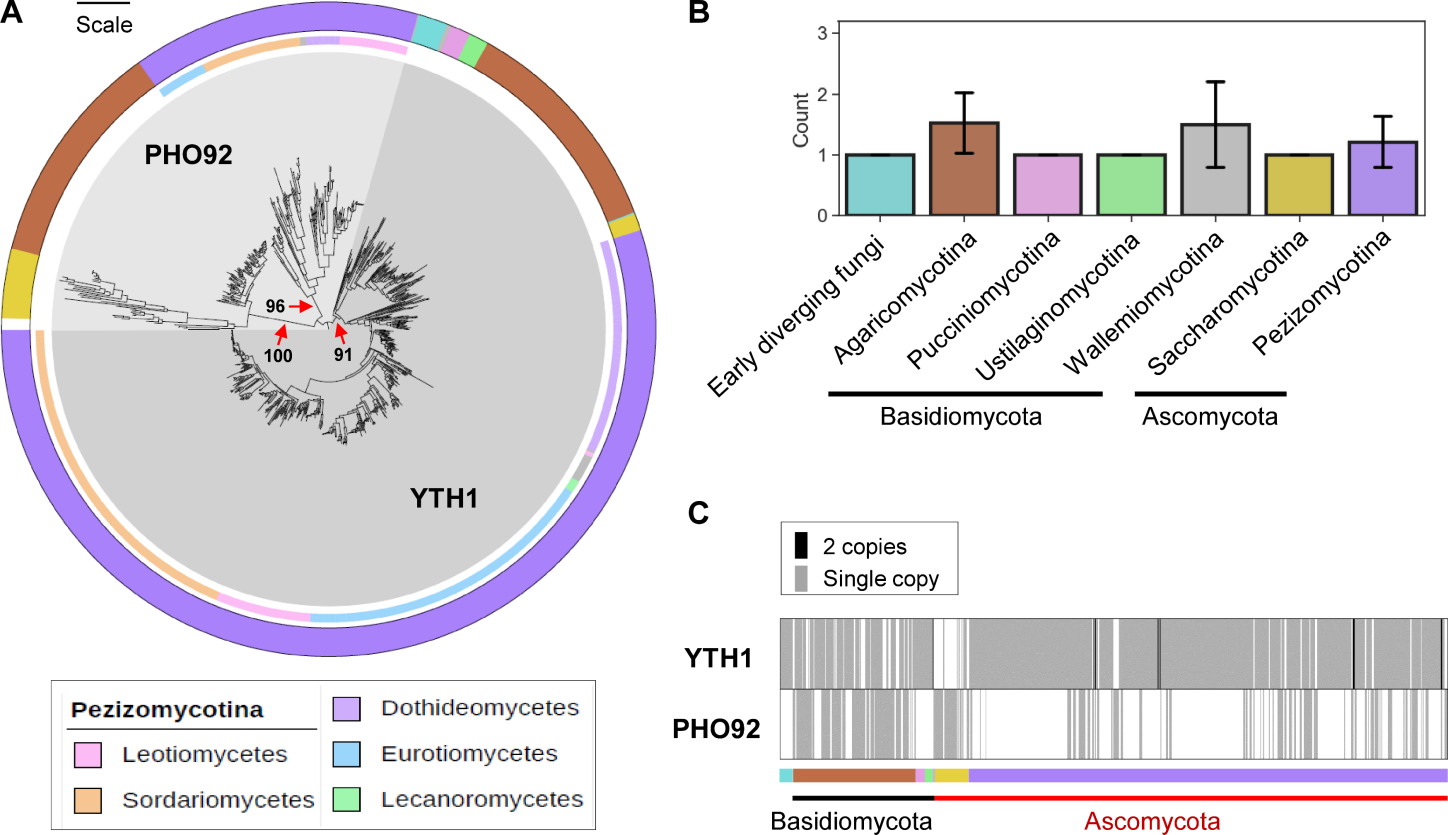
Phylogeny of potential m^6^A readers in fungi. (**A**) The maximum-likelihood phylogeny was estimated from 868 sequences of YTH domain-containing proteins identified in 695 fungal species. Red arrows indicate highly supported clades representing PHO92 (Agaricomycotina and Saccharomycotina), PHO92 (Pezizomycotina), and YTH1 families. The branch lengths in the tree reflect the amount of evolutionary change, with the scale indicating 1.0 amino acid sequence substitution per site. The outer color strip represents subphyla, with each color indicating a distinct taxonomic group as shown in (**B**), and the inner color strip represents fungal class within the subphylum Pezizomycotina, with each color indicating a distinct taxonomic group as shown in the inset box. (**B**) Phyletic distribution of YTH domain-containing proteins in fungi. (**C**) Copy number variation of potential m^6^A readers in fungi. The color strip represents different fungal taxa, with each color indicating a distinct taxonomic group as shown in Fig. 2B.

Among RNA-binding proteins specifically recognizing and binding to m^6^A, the best studied readers are YTH domain-containing proteins. PHO92 was found as the only protein containing the YTH domain in the budding yeast, while animals can have up to five such proteins and plants can have more than 10. A recent study revealed target transcripts of PHO92 and its important roles during meiosis in budding yeast (37). However, little is known about m^6^A readers in other fungal taxa. Therefore, we searched for proteins containing a YTH domain (PF04146) in the UniProt database (accessed on 21 May 2023) and found 2,029 fungal sequences. To shorten the list of potential m^6^A readers, entries with irrelevant protein names (e.g., DNA repair protein RAD51) were excluded, and protein sequences below 300 or above 1,000 amino acids in length were filtered out. After manual curation, we reconstructed a phylogenetic tree for the final list of 868 YTH domain-containing proteins in 695 diverse fungal species (Fig. 3A; see Fig. S1 for bootstrap values and protein IDs). The tree was rooted to YTH domain-containing proteins outside of fungi, including human YTHDF2. The tree was composed of three main clades that were strongly supported by high bootstrap values. One clade contained PHO92 homologs from species belonging to Saccharomycotina and Basidiomycota, as well as YTH domain-containing proteins found in mammals, an insect, and a plant. Another clade exclusively comprised species from Pezizomycotina and was placed sister to the PHO92 clade. The third clade, here dubbed YTH1, consisted of early-diverging fungi and species from Basidiomycota and Ascomycota, displaying a topology that largely matched the species phylogeny. Approximately half of the Agaricomycotina species (67 out of 128) and one-fifth of the Pezizomycotina species (98 out of 498) examined in this study were found to possess both PHO92 and YTH1 while early-diverging fungi and species belonging to Pucciniomycotina, Ustilaginomycotina, and Wallemiomycotina, were found to have only PHO92 (Fig. 3B and C). While the majority of species belonging to Saccharomycotina (30 out of 37) were found to possess PHO92, there were several species including *Yarrowia lipolytica* (a lipid-producing yeast) that lacked PHO92 and instead exhibited the presence of YTH1 (Fig. 3C; Table S1).

**Fig 4:**
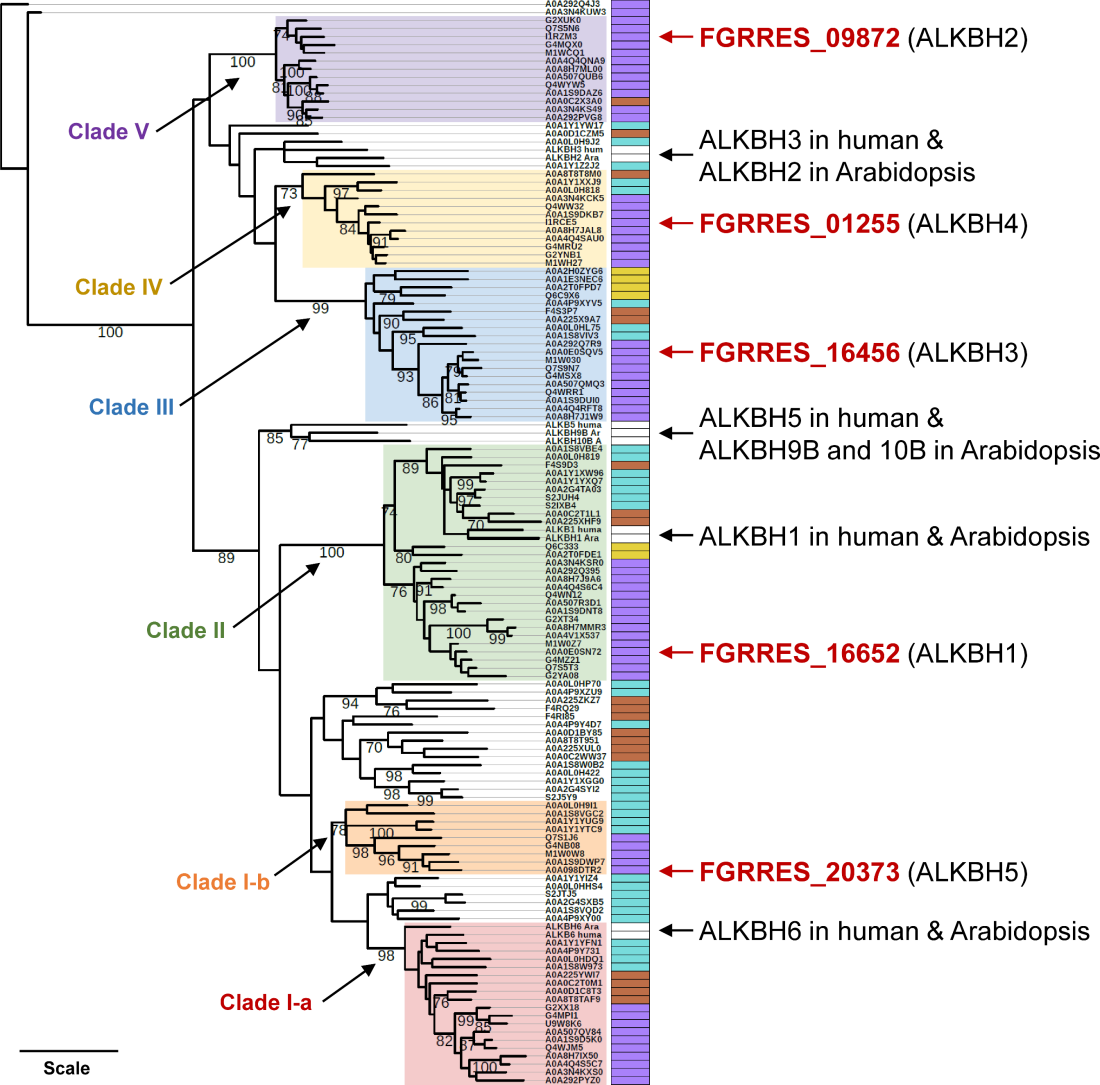
Phylogeny of potential m^6^A erasers in fungi. The maximum-likelihood phylogeny was estimated from 125 sequences of 2OG-Fe(II) dioxygenase AlkB-like domain-containing proteins identified in 27 selected fungal species. The color strips on the right side of leaves indicate different fungal taxa: purple—Pezizomycotina, yellow—Saccharomycotina, blown—Basidiomycota, cyan—early-diverging fungi, white—human or *Arabidopsis thaliana*. Highly supported clades each containing an ALKBH identified in *F. graminearum* were shaded with different colors. Bootstrap values of greater than 70% were shown. Branch lengths are proportional to the inferred amount of evolutionary change, and the scale represents 1.0 amino acid sequence substitutions per site.

2OG-Fe[II] dioxygenase AlkB-like domain-containing proteins were known to play roles as m^6^A erasers by removing the methyl group from m^6^A in mammals and plants (26, 48–50). However, there has been no information on AlkB homologs (ALKBHs) in fungi. Thus, we searched for protein sequences containing a 2OG-Fe(II) dioxygenase superfamily domain in the genome of *F. graminearum* strain PH-1. We identified five genes harboring a 2OG-Fe(II) dioxygenase domain (PF13532) with an *E*-value of smaller than 10^−10^ (gene ID: FGRRES_01255, FGRRES_09872, FGRRES_16456, FGRRES_16652, and FGRRES_20373) (Table 1). 2OG-Fe(II) dioxygenases are involved in diverse cellular processes, such as demethylation of DNA/RNA and modification of histone proteins, as well as secondary metabolite production. To get insights into evolutionary relationships of different types of ALKBH, we reconstructed a phylogenetic tree of ALKBH proteins found in 27 selected fungi, including several functionally characterized ALKBH proteins in humans and Arabidopsis (Fig. 4). Early-diverging fungi, such as *Basidiobolus meristosporus* and *Spizellomyces punctatus*, possessed 10 and 9 2OG-Fe(II) dioxygenases, whereas yeasts that belong to Saccharomycotina tend to have only one or two (Table S2). ALKBH2 (FGRRES_09872), ALKBH3 (FGRRES_16456), and ALKBH4 (FGRRES_01255 were closely related to human ALKBH3 and Arabidopsis ALKBH2, which are responsible for repairing DNA damage caused by alkylation (51, 52). ALKBH5 (FGRRES_20373) was placed sister to the clade containing human ALKBH6, which plays a role in DNA repair (53, 54). ALKBH1 (FGRRES_16652) formed a well-supported clade with human and Arabidopsis ALKBH1s, known for their versatile functions in removing methyl groups from DNA/RNA, as well as modifying histone H2A (55–57). It was shown that human ALKBH5 and Arabidopsis ALKBH9B and ALKBH10B demethylate m^6^A (48, 58–60). These m^6^A erasers in mammals and plants formed a clade ancestral to clades including ALKBH1 and ALKBH5 in *F. graminearum* (Fig. 4).

**TABLE 1:** Putative m^6^A factors found in *F. graminearum*

Name	ID (FGRRES)	AA	Pfam	E-value	Putative function
MTA1	06225	333	PF05063	1.2e-34	writer
WTAP^*a*^	01626	266	PF17098	0.069	writer
VIRN^*a*^	06249	143	PF15912	0.073	writer
YTH1	01159	613	PF04146	9.9e-66	reader
ALKBH1	16652	346	PF13532	3.5e-30	eraser
ALKBH2	09872	933	PF13532	1e-29	eraser
ALKBH3	16456	443	PF13532	5.6e-35	eraser
ALKBH4	01255	326	PF13532	2.8e-28	eraser
ALKBH5	20373	228	PF13532	6.2e-15	eraser

^a^ *E*-value is greater than 10^−5^.

### Functional characterization of putative m^6^A factors in *F. graminearum*

**Fig 5:**
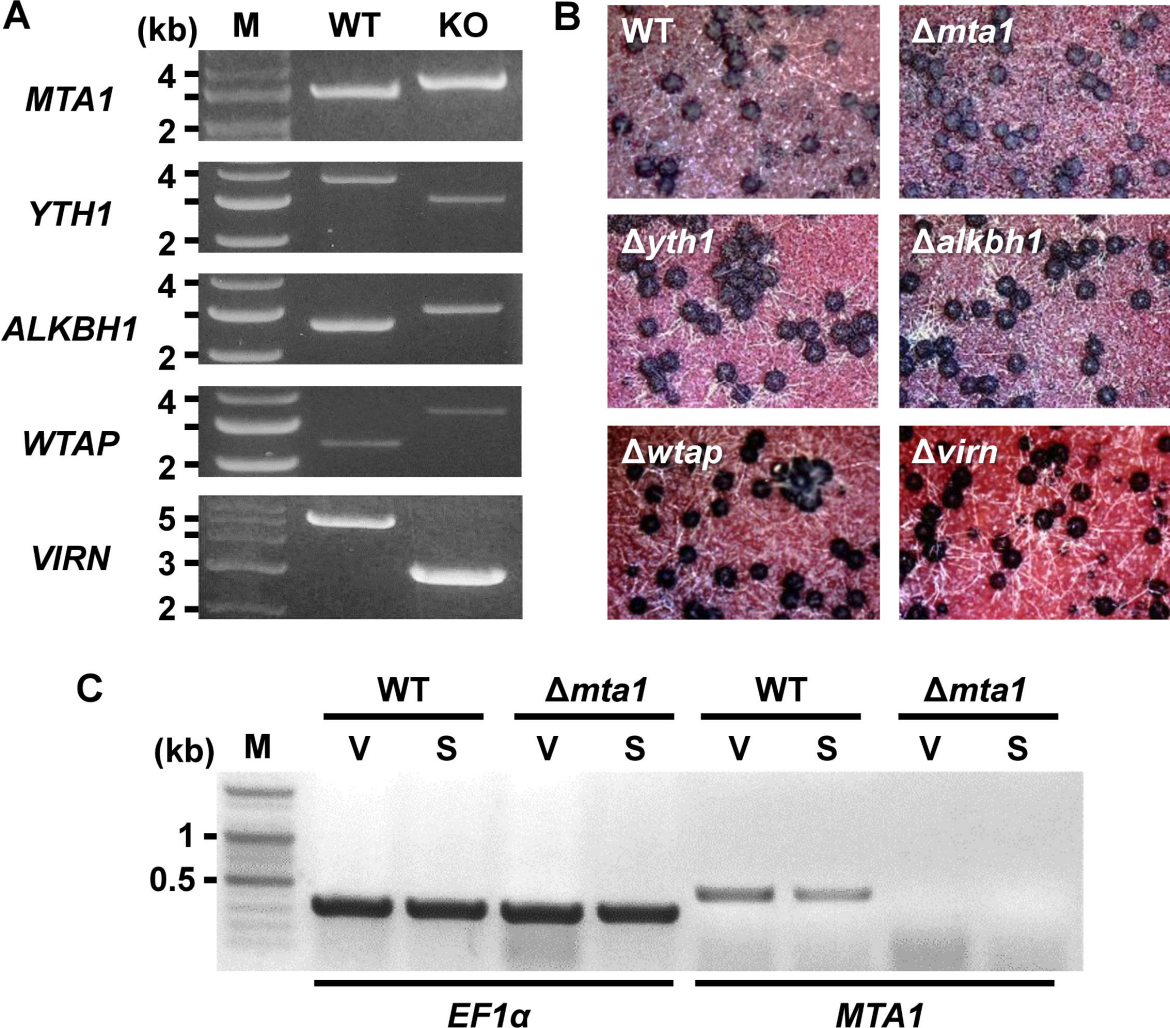
Generation of knockout mutants lacking potential m^6^A factors in *F. graminearum*. (**A**) Diagnostic PCRs confirmed homologous integration of the split marker constructs to the target gene loci. Note the PCR band size between the WT and knockout strains (KO) due to the gene replacement. M—1 kb DNA ladder. (**B**) Perithecia production of the WT and knockout strains grown on carrot agar media. Photographs were taken with a dissecting microscope 6 days after induction of sexual development (20× magnification). (**C**) RT-PCR analyses of the WT and Δ*mta1* strains. Since *MTA1* lacks introns, distinguishing RNA expression from possible genomic DNA contamination in RT-PCR analysis was challenging. To verify RNA integrity, a primer set was designed for amplifying *EF1α*, including two introns. Notably, no band corresponding to genomic DNA amplicon for the EF1α reference gene was observed (658 bp for gDNA, 356 bp for mRNA), indicating the absence of genomic DNA contamination. Expression of *MTA1* was confirmed in the WT, whereas no discernible band was observed in the Δ*mta1* strain. The samples labeled V were collected before sexual induction (i.e., vegetative growth stage), and samples labeled S were collected 6 days after sexual induction (i.e., sexual growth stage). M—100 bp DNA ladder.

To examine the functions of m^6^A factors in *F. graminearum* during sexual development, we conducted a search for Pfam domains associated with m^6^A writer, reader, and eraser, and discovered several potential m^6^A factors (Table 1). Among them, *F. graminearum* possessed genes homologous to *MTA1* and *YTH1*, along with the previously mentioned five *ALKBHs*. In addition, we identified two genes that encoded hypothetical proteins harboring PF17098 for WTAP and PF15912 for VIRN, respectively, with *E*-values of greater than 10^−5^ (Table 1). Despite the low sequence similarity to *WTAP* and *VIRN* found in fungi, we included these potential m^6^A writers in the knockout study because their expression levels were observed to increase during sexual development (see Fig. 7 in the section “Roles of MTA1 in conidial germination and hyphal growth”). We individually deleted m^6^A factors in *F. graminearum* strain PH-1 [wildtype (WT)]. The authenticity of the resulting knockout mutants was verified by diagnostic PCRs (Fig. 5A). The knockouts displayed normal growth and produced perithecia, which are characterized by their dark purple coloration and flask-shaped or spherical structures (Fig. 5B). Morphologies of ascospores (the products of meiosis) enveloped in sac-like structures called asci were also normal in the knockouts, indicating that m^6^A factors did not affect sexual development in *F. graminearum* (Fig. S2).

Since m^6^A writer, *IME4,* in the budding yeast, plays a crucial role in initiating sexual development, we carefully examined the genotype of Δ*mta1*. Although genotyping confirmed the deletion of *MTA1* in Δ*mta1*, we were intrigued to find that a PCR band specific to *MTA1* could still be amplified from Δ*mta1*. This suggested that Δ*mta1* is heterokaryotic, indicating that it still contains one or more “wild-type” nuclei. To obtain homokaryons, we performed additional single-spore isolations, using Δ*mta1*, and examined 10 single-spored isolates derived from Δ*mta1*. Diagnostic PCR analysis confirmed the deletion of *MTA1* in all 10 isolates; yet, a fragment of *MTA1* was still amplified from these isolates (Fig. S3). The expression levels of *MTA1* were markedly reduced in Δ*mta1*, suggesting a lower abundance of wild-type nuclei compared to the WT (Fig. 5C). This observation was further supported by RNA-seq analysis. When examining the mapped reads on the *MTA1* locus in both WT and Δ*mta1*, we found a decrease in the number of reads mapped to the *MTA1* locus in Δ*mta1* compared to the WT (Fig. S3). The presence of RNA-Seq reads mapping to the *MTA1* locus, which should have been absent in homokaryotic Δ*mta1*, served as additional proof for the heterokaryotic nature of Δ*mta1* and indicated that Δ*mta1* is, in fact, a knockdown strain. Consequently, we will refer to Δ*mta1* as the *MTA1*-KD1 strain hereafter.

### MTA1 as an m^6^A writer in *F. graminearum*

**Fig 6:**
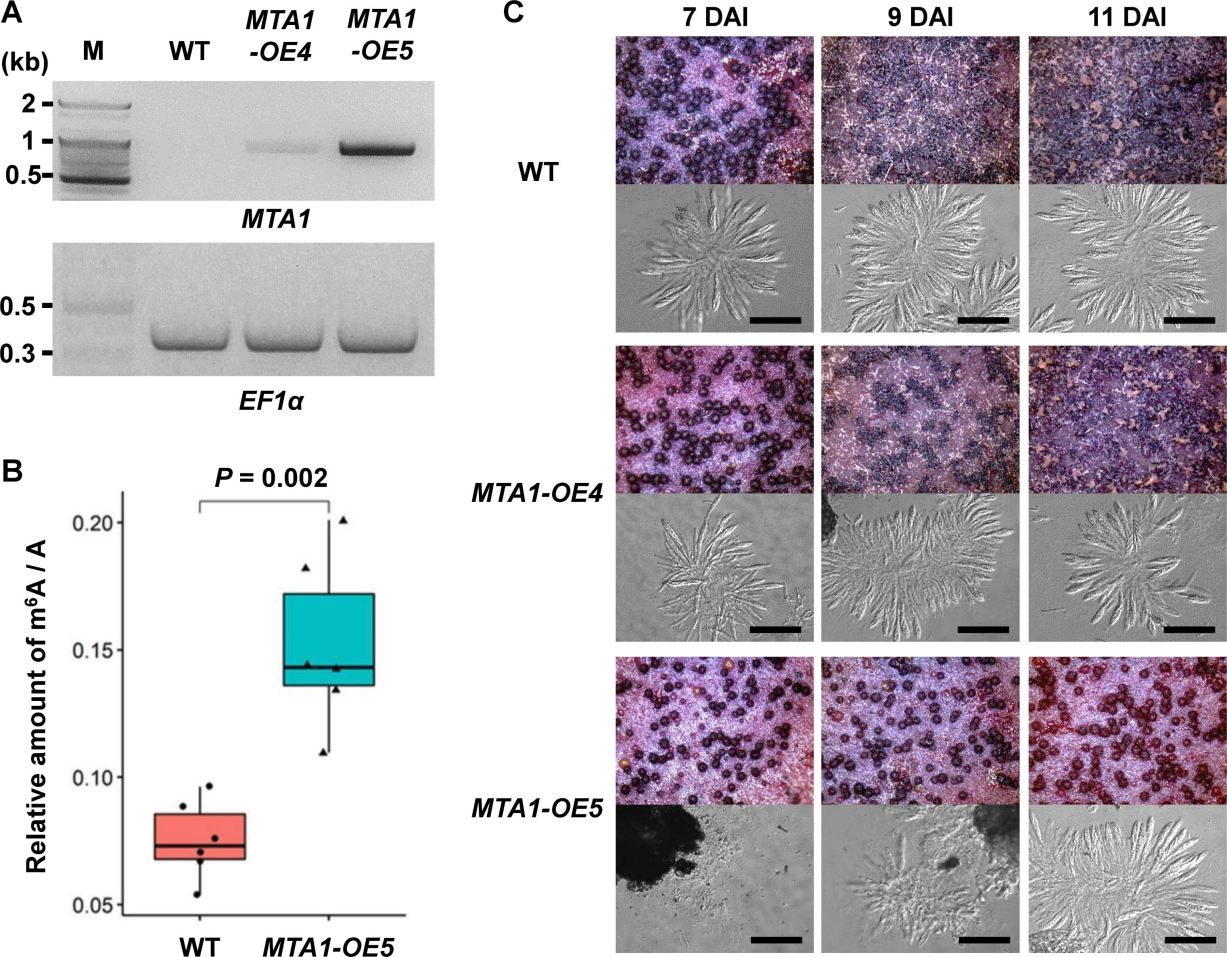
Overexpression of the m^6^A writer *MTA1*. (**A**) RT-PCR analyses of the WT and *MTA1*-overexpressing strains (*MTA1*-OE4 and *MTA1*-OE5). Since *MTA1* lacks introns, distinguishing RNA expression from possible genomic DNA contamination in RT-PCR analysis was challenging. To verify RNA integrity, a primer set was designed for amplifying *EF1α*, including two introns. Notably, no band corresponding to genomic DNA amplicon for the EF1α reference gene was observed (658 bp for gDNA, 356 bp for mRNA), indicating the absence of genomic DNA contamination. Overexpression of *MTA1* was confirmed in the *MTA1*-OE4 and *MTA1*-OE5 strains. Note that *MTA1* was significantly overexpressed in the *MTA1*-OE5 strain, compared to the WT, from which no discernible band was observed at the PCR cycle of 30. M—100 bp DNA ladder. (**B**) m^6^A methylation level in RNA extracted from the WT and MTA1-OE5 strains. Mann-Whitney U test was performed to compare the means of the ratio for m^6^A to A between the WT and *MTA1*-OE5 strains. Box and whisker plots indicate the median, interquartile range between the 25th and 75th percentiles (box), and 1.5 interquartile range (whisker). (**C**) Perithecia production of the WT, MTA1-OE4, and *MTA1*-OE5 strains grown on carrot agar media (upper panels, 20× magnification). Photographs were taken with a dissecting microscope at the indicated days after induction of sexual development (DAI). Squash mounts of perithecia were observed with a compound microscope (bar = 50 µm). Mature ascospores were observed in the WT and MTA1-OE4 strains as early as 7 DAI, whereas in the MTA1-OE5 strain, it was not until 11 DAI that ascospore formation became evident.

Extensive efforts were unsuccessful in obtaining knockouts completely lacking *MTA1* (Fig. S3), presumably due to its indispensable role in *F. graminearum*. To get better insight into the roles of m^6^A RNA methylation in *F. graminearum*, we generated strains overexpressing *MTA1*. RT-PCR analysis confirmed that two strains, *MTA1*-OE4 and *MTA1*-OE5, exhibited overexpression of *MTA1* (Fig. 6A). It was notable that *MTA1* was overexpressed to a greater extent in the *MTA1*-OE5 strain, compared to *MTA1*-OE4. Both semi-quantitative RT-PCR and RNA-seq analyses indicated that the expression level of the *MTA1*-OE5 strain was approximately 100 times greater than that of the WT (Fig. S4). The WT and *MTA1*-OE4 strains produced normal asci containing mature ascospores 6 days after the induction of sexual development, while it was not until 11 days after the induction that the *MTA1*-OE5 strain produced fully developed asci (Fig. 6B). The difference in the observed phenotypes in the two *MTA1*-overexpressing strains could likely be attributable to the variation in the degrees of *MTA1* expression, and thus the *MTA1*-OE5 strain which displayed substantial overexpression of *MTA1* was used for further analysis and investigation. To determine whether MTA1 is required for m^6^A RNA methylation in *F. graminearum*, we compared total m^6^A RNA methylation levels between the WT and *MTA1*-OE5 strains. The amount of m^6^A RNA in WT was 0.075% ± 0.015% of the total RNA, which was approximately half of that in the *MTA1*-OE5 strain (0.152% ± 0.033% of the total RNA), which indicated that the m^6^A RNA methylation level was significantly increased in the *MTA1*-OE5 strain compared to the WT (Fig. 6C).

### Roles of MTA1 in conidial germination and hyphal growth

**Fig 7:**
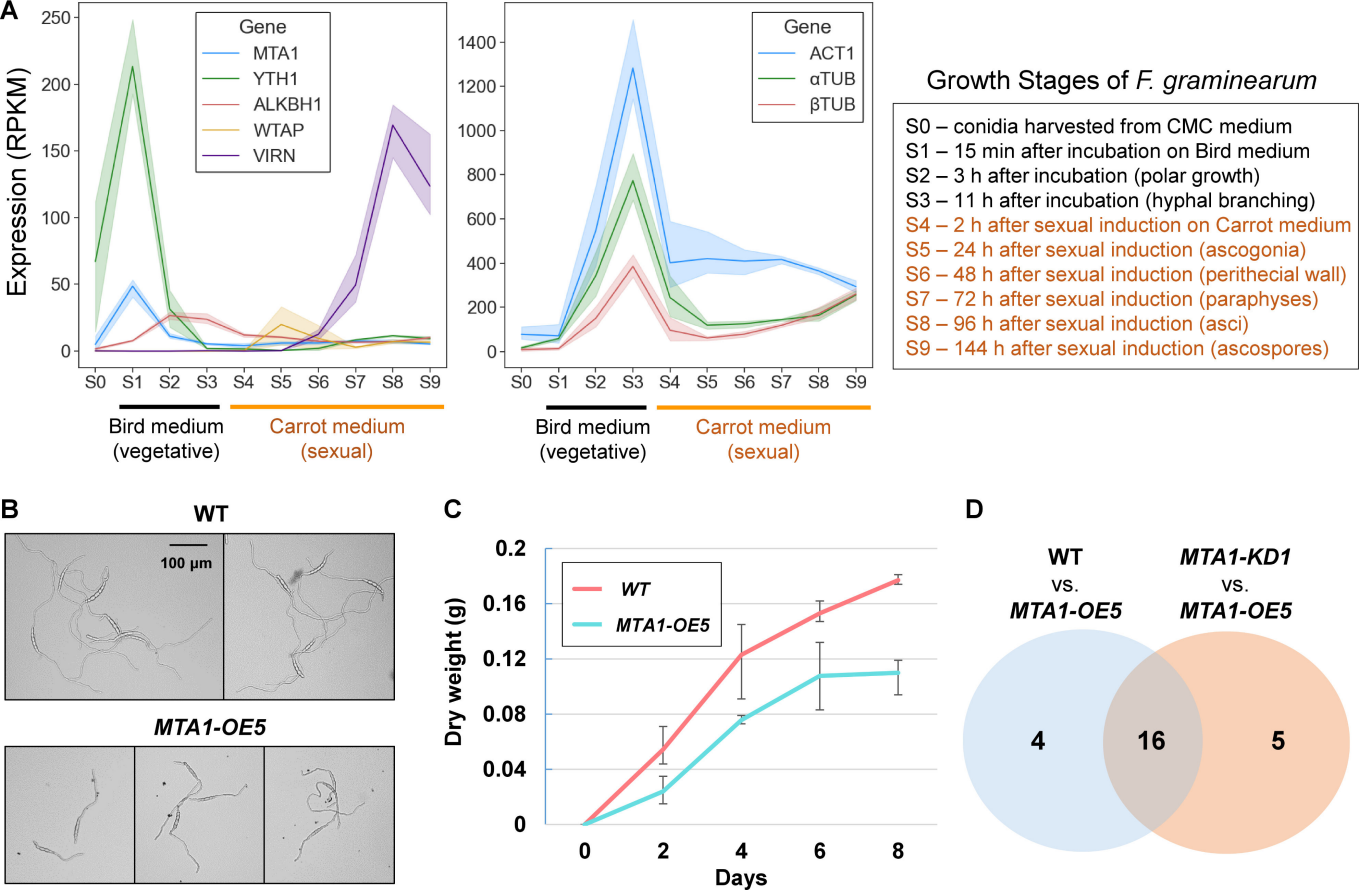
Conidial germination affected by the m^6^A writer *MTA1*. (**A**) Gene expression profiles of potential m^6^A factors (left panel) and housekeeping genes (right panel) in *Fusarium graminearum*. Average values for reads per kilobase per million mapped reads (RPKM) values for three replicate samples were plotted. Bands surrounding the line plots indicate 95% confidence intervals of the means. The *x*-axis are different growth stages of *F. graminearum* (S0–S9). See the right box for the description of vegetative and sexual growth stages. Gene ID for potential m^6^A factors are ALKBH1 (FGRRES_16652), MTA1 (FGRRES_06225), VIRN (FGRRES_06249), and WTAP (FGRRES_01626). Housekeeping genes examined here are α-tubulin (αTUB, FGRRES_00639), β-tubulin (β TUB, FGRRES_09530), and actin 1 (ACT1, FGRRES_07735). (**B**) Conidia germination and polar growth of hyphae in quarter-strength potato dextrose broth (q-PDB) medium. Photos were taken 16 h after incubation. Note that shorter hyphae germinated from macroconidia of the MTA1-OE5 strain, compared to the WT strain. (**C**) The dry weight of mycelia grown in q-PDB medium was measured at 2 days interval. (**D**) The numbers of differentially expressed genes in fresh macroconidia harvested from carboxymethylcellulose (CMC) medium between the WT and MTA1-OE5 strains, and between the MTA1-KD1 and MTA1-OE5 strains.

In previous studies, we obtained transcriptome data for conidial germination on the Bird agar medium (61), as well as for perithecial development on the Carrot agar medium (62). By integrating these data sets with the RNA-seq data obtained in the present study (freshly harvested conidia), we conducted a comprehensive analysis of expression level changes in m^6^A factors throughout the life cycle of *F. graminearum*, encompassing various stages of vegetative growth and perithecial development (Fig. 7). The m^6^A writer MTA1 and a potential m^6^A reader YTH1 demonstrated a synchronized expression pattern, as indicated by a high Pearson correlation coefficient of 0.94. Their expression levels exhibited a significant increase during stage 1 (S1, 15 minutes after incubation of conidia on Bird agar medium), followed by a decline during hyphal growth (S2 and S3), and remained at a basal level during perithecial development (S4–S9). Interestingly, the expression levels of ALKBH1 and ALKBH5, the two most probable m^6^A erasers in *F. graminearum*, reached their peak at stage 2 (S2, 3 h after incubation), at which conidia germinated and hyphae started to extend (Fig. S5). During active hyphal growth at stage 3 (S3, 11 h after incubation), the major components of the fungal cytoskeleton, actin (FGRRES_07735) α-tubulin (FGRRES_00639), and β-tubulin (FGRRES_09530) that play a crucial role in polarity establishment, maintenance, and polar growth, displayed the greatest induction during the life cycle of *F. graminearum*.

Taking into account the expression profile, we proceeded to investigate the phenotypic characteristics of the MTA1-OE5 strain during conidial germination and hyphal growth. In contrast to the WT, the hyphal tips did not exhibit elongation in an overnight culture of the MTA1-OE5 strain cultivated in quarter-strength potato dextrose broth (q-PDB) medium (Fig. 7B). Furthermore, in the MTA1-OE5 strain growing on potato dextrose agar (PDA) medium, the hyphae at the leading edge displayed a tendency to have fewer branches compared to the WT (Fig. S6). Next, we conducted measurements of the total biomass of the WT and MTA1-OE5 strains cultured in q-PDB medium for a duration of 8 days. The MTA1-OE5 strain exhibited a slower growth rate compared to the WT, particularly during the initial 2 days of cultivation (Fig. 7C). To investigate genes responsible for the delayed conidial germination observed in the MTA1-OE5 strain, we performed differential gene expression analyses between the WT, MTA1-KD1, and MTA1-OE5 strains, using RNA-seq data obtained from freshly harvested conidia (stage 0, S0). In this analysis, we identified a total of 20 differentially expressed (DE) genes [fold change > 4 at a false discovery rate (FDR) of 5%], when comparing the WT and MTA1-OE5 strains, as well as 21 DE genes when comparing the MTA1-KD1 and MTA1-OE5 strains (Fig. 7D). Notably, 16 genes were found to be commonly differentially expressed in both comparisons, including MTA1. There were no DE genes observed between the WT and MTA1-KD1 strains (Fig. S7). Among the DE genes, we found that 17 genes were transcriptionally upregulated, and eight genes were downregulated in the MTA1-OE5 strain (Table 2). The DE genes included nine genes encoding hypothetical proteins without any predicted protein domain and 16 functionally annotated genes encoding two transcription factors, two transporters, and diverse enzymes. FGRRES_00725 and FGRRES_05926 encoding GAL4-type transcription factors were significantly downregulated in the MTA1-OE5 strain, while FGRRES_02139 and FGRRES_04188 encoding an ABC multidrug transporter and major facilitator superfamily, respectively, were highly upregulated in the MTA1-OE5 strain (Table 2). In consistent with the expression pattern of MTA1, the expression levels of two transporters were elevated at S1 and reduced thereafter. For two transcription factors, the expression levels of FGRRES_05926 and FGRRES_00725 began to decline at S2 and S1, respectively, and remained basal, suggesting that their roles in early stages of conidial germination and hyphal growth.

**TABLE 2:** Differentially expressed genes in the *MTA1*-overexpressing strain

Gene ID (FGRRES)	FC^*a*^	FDR^*b*^	Predicted function
11026	−5.2	0.0212	Nonribosomal peptide synthetase, malonichrome
16138	−6.0	0.0212	Hypothetical protein
11158	−8.1	0.0212	Amidohydrolase family
16091^c^	−4.3	0.0212	Hypothetical protein
17136	5.2	0.0212	Superfamily I DNA and/or RNA helicase
03600	8.2	0.0212	Hypothetical protein
00251	−5.0	0.0212	Probable galactose oxidase precursor
13979	5.5	0.0233	Acetyltransferase (GNAT) domain
04188^*c*^	4.0	0.0233	Major facilitator transporter
11412	5.5	0.0233	Hypothetical protein
12820	6.9	0.0245	Hypothetical protein
05926	−3.8	0.0245	GALl4-type MHR TF (without a zinc cluster domain)
13561	4.7	0.0245	FAD-dependent oxidoreductase
10598^*c*^	6.6	0.0245	Hypothetical protein
02139	5.7	0.0245	ABC transporter
11413	6.9	0.0255	CoA-transferase family III domain-containing protein
06225	7.0	0.0306	MTA1, m6A writer
00725^c^	−4.2	0.0499	GAL4-type MHR TF (with a zinc cluster domain)
11736	4.6	0.0499	Hypothetical protein
05829	4.9	0.0499	Prolyl oligopeptidase family
03319^*d*^	4.6	0.0597	AAA family ATPase
11474^*d*^	5.3	0.0901	Nucleotidyltransferase superfamily, GrpB domain-containing
15648^*d*^	5.1	0.0901	Hypothetical protein
08659^*d*^	2.8	0.1125	Hypothetical protein
10446^*d*^	−4.2	0.1479	Pyruvate decarboxylase

^a^ log2-transformed fold change in the *MTA1*-overexpressing strain.

^b^ False discovery rate.

^c^ Differentially expressed genes only in comparison between the wild-type and *MTA1*-overexpressing strain.

^d^ Differentially expressed genes only in comparison between the Δ*MTA1* and *MTA1*-overexpressing strain.

## DISCUSSION

### Roles of m^6^A writer in filamentous fungi

In the phylum Ascomycota, the roles of m^6^A writers in budding yeasts (Saccharomycotina) and filamentous fungi (Pezizomycotina) may have evolved independently. Species belonging to the Pezizomycotina possess only a single m^6^A writer *MTA1*, a homolog of *METTL4*, while species belonging to the Saccharomycotina have two m^6^A writers, *IME4* and *KAR4* that are homologs of *METTL3* and *METTL14*, respectively. In budding yeast, *IME4* and *KAR4* are crucial for the initiation of meiosis for ascospore formation (29, 63). However, the m^6^A writer *MTA1* is dispensable for perithecial development and ascospore production in filamentous fungi, *F. graminearum* and *M. oryzae* (46). The relative abundance of m^6^A in *M. oryzae* (0.069% ± 0.003%) is comparable to that in *F. graminearum* (0.075 ± 0.015%) (47), suggesting that the degree of m^6^A levels of total mRNA is similar in these two filamentous fungi. In the rice blast fungus *M. oryzae*, a knockout strain lacking *MTA1* showed a defect in appressorium formation during the infection process on the epidermal cells of rice (47). Expression levels of autophagy-related genes in *M. oryzae* were changed according to the degree of m^6^A level, which is important for normal appressorium formation. Although the homozygous Δ*mta1* strain in *M. oryzae* was viable, our extensive efforts to obtain homozygous Δ*mta1* were unsuccessful in *F. graminearum*, suggesting its essential role. Given the significant impact on conidial germination and hyphal growth observed in the *MTA1*-overexpressing strain in *F. graminearum*, the role of *MTA1* appears to have diverged in the two plant pathogenic fungi.

Most importantly, *F. graminearum* does not form appressoria and utilizes distinct strategies to penetrate the epidermal cells of the host plants. Both *F. graminearum* and *M. oryzae* belong to the Sordariomycetes but fall into different orders, Hypocreales and Magnaporthales, respectively (64). RNA-seq analyses indicated significant divergence in gene expression including autophagy-related genes during conidial germination and infection processes between *F. graminearum* and *M. oryzae* (61). The m^6^A writers *IME4* and *KAR4* were crucial for the initiation of the sexual cycle and the formation of ascospores in the budding yeast. However, the m^6^A writer *MTA1* does not appear to be responsible for perithecial development and ascospore production in *F. graminearum* and *M. oryzae*. Although the *MTA1*-OE5 strain exhibited delayed perithecial development, it produced normal ascospores. In the Pezizomycotina, A-to-I RNA editing, one of the eukaryotic RNA modifications, is crucial for sexual development and ascospore formation (43–45, 65–67).

### Evolutionary perspectives of m^6^A factors in fungi

Several ancestral traits observed in early-diverging fungi are shared with metazoans or unicellular opisthokonts, which have been subjected to extensive parallel loss across the Dikarya lineages (68, 69). As with metazoans, many species of early-diverging fungi and species that belong to the Pucciniomycotina (the earliest-diverging subphylum within Basidiomycota) possess all three m^6^A writers, IME4, MTA1, and KAR4. These suggested that budding yeasts (Saccharomycotina) may have lost the m^6^A writer MTA1. Conversely, species belonging to Pezizomycotina, which entirely consists of filamentous fungi, may have lost the m^6^A writers IME4 and KAR4. The phyletic distribution of m^6^A writers in fungi suggested that Pezizomycotina species including *F. graminearum* and *M. oryzae* have likely embraced m^6^A machineries, such as *MTA1*, as a means to regulate filamentous growth and facilitate host penetration. This adoption of m^6^A machinery may have played a crucial role in the development and maintenance of the filamentous morphology that is characteristic of these fungi, allowing them to effectively colonize and interact with their respective hosts.

In contrast to well-characterized yeast m^6^A methyltransferase complexes including *IME4* and *KAR4* m^6^A writers, our understanding of m^6^A machinery in filamentous fungi is still limited. Despite our functional characterization of potential *WTAP* and *VIR1* homologs in *F. graminearum*, it is unlikely that these represent genuine homologs. This is because the homologs of *WTAP*/*MUM2* and *VIR1* are missing in species within all the Pezizomycotina species. The failure to identify homologs of *WTAP* and *VIR1* in our study is likely due to significant sequence divergence of these m^6^A factors within the Pezizomycotina. The sequence differences might have hindered their detection using conventional homology search methods, indicating that these m^6^A factors in Pezizomycotina may have undergone substantial changes in the primary amino acid sequences. Until recently, the existence and function of *VIR1* in the budding yeast had remained unknown, but through the application of protein folding prediction tools, its identity was revealed (32). Recent advancements in protein 3D structure prediction tools to uncover hidden homologs (70, 71) hold promise for identifying homologous members of the m^6^A methyltransferase complexes in Pezizomycotina species. Alternatively, it is possible that components of m^6^A machinery associated with MTA1 are completely distinct from those found in the yeast m^6^A methyltransferase complex associated with IME4 and KAR4. In this case, it will be necessary to identify components that interact with MTA1 *via* co-immunoprecipitation.

In addition, it is worth mentioning, considering the number of putative m^6^A writers and m^6^A erasers found in early-diverging fungi and species from the Pucciniomycotina (up to five m^6^A writers in a species), not many m^6^A readers containing an YTH domain were identified, suggesting that there may be different types of RNA-binding proteins that recognize m^6^A modification. It would be interesting to study the possibly divergent roles of m^6^A factors in these relatively understudied fungal taxa.

### Expression levels of m^6^A factors throughout the life cycle of *F. graminearum*

The levels of *MTA1* and *YTH1* expression were significantly elevated at 15 minutes after placing conidia on the Bird medium (S1) and then decreased sharply to a basal level during polar growth and hyphal branching (S2–S3). These findings suggest that the potential m^6^A writer and m^6^A reader likely have a role during the initial stage of conidial germination. In line with this hypothesis, we observed slower conidial germination and less hyphal branching in the *MTA1*-OE5 strain. Although m^6^A demethylase activities of *ALKBH* genes have never been confirmed in fungi, the three putative m^6^A erasers, *ALKBH1*, *ALKBH4*, and *ALKBH5*, were induced during polar growth (S2) or hyphal branching (S3). These possible m^6^A erasers might be involved in maintaining low m^6^A levels during active vegetative growth. The significant increase in expression of actin and tubulin genes at S2–S3 reflected extensive hyphal growth and branching. Specifically, the expression level of *ALKBH4* reached its peak at S0 (fresh conidia) but experienced a sharp decline at S1 (the initial stage of conidial germination). However, it was gradually induced during polar growth and hyphal branching stages (S2-S3), exhibiting an opposite expression pattern compared to *MTA1* and *YTH1*. Although we did not observe any phenotypic changes in the knockout mutant lacking *ALKBH1*, it would be possible that other *ALKBH* genes may be involved in the regulation of conidial germination and hyphal growth by maintaining low m^6^A level in *F. graminearum*. Among the five putative m^6^A erasers, *ALKBH2* showed a dramatic increase in expression during the ascospore formation. These expression dynamics of m^6^A factors highlight the complex regulation and involvement of m^6^A factors throughout different stages of the *F. graminearum* life cycle.

### Concluding remarks

The largest subphylum Pezizomycotina in fungi underscores their importance and their complex interactions with humans, encompassing a wide range of fungi that have significant influences on humans, both negative and positive. Examples include the opportunistic human pathogen *Aspergillus fumigatus* and the dermatophyte *Trichophyton rubrum*, as well as many plant pathogenic fungi causing serious diseases on our staple crops, such as *F. graminearum* and *M. oryzae*. The Pezizomycotina also includes several ecologically significant species, encompassing those that play crucial roles in wood and litter decay processes, as well as those that form symbiotic associations with other organisms, including lichens. In filamentous fungi, the m^6^A writer *MTA1* appears to have evolved to have important roles in adapting to diverse ecological niches, particularly in relation to filamentous growth. To identify target genes of *MTA1* that caused delayed conidial germination and slower hyphal growth in the *MTA1*-OE5 strain, we are currently investigating potential m^6^A sites in transcripts, using an Oxford Nanopore direct RNA sequencing technology.

## MATERIALS AND METHODS

### Phylogenetic analyses of m^6^A factors in fungi

We downloaded protein sequences from the UniProt database (https://www.uniprot.org/) that possessed pfam domains associated with m^6^A factors. These domains include PF05063 (MT-A70), PF04146 (YTH), PF13532 (ALKB), PF17098 (WTAP/MUM2), and PF15912 (Virilizer, N-terminal). However, the initial lists of potential m^6^A factors contained duplications and likely misannotated sequences. To address this issue, we conducted a manual inspection of the lists, excluding sequences that were either too short or too long, as well as sequences with small E -values, applying specific thresholds (e.g., E -value > 10^–5^). Whenever a sequence was removed from the lists, we also eliminated all entries from the corresponding species to ensure an accurate estimation of the number of m^6^A factors per species. The sequences for each pfam domain were aligned using MAFFT (v7.310) with the “auto” setting (72). Poorly aligned regions of the resulting multiple sequence alignment were trimmed, using the Trimal program, with the parameter setting “–gappyout” (73). To determine the best protein substitution model for each pfam domain, we used a perl scrip that can be found on the following website (https://github.com/stamatak/standard-RAxML/blob/master/usefulScripts/ProteinModelSelection.pl). We selected protein substitution models GAMMALG for m^6^A writers, GAMMAJTTF for m^6^A readers, GAMMALGF for ALKBHs and VIRN, and GAMMAJTT for WTAP. Maximum likelihood trees were constructed using the RAxML program (v8.2) (74). The outgroup was set to the homologue of m^6^A factors in Arabidopsis thaliana and nodal supports were evaluated by 1,000 bootstrap replications.

### Genetic transformation for gene deletion and overexpression

To generate gene deletion mutants in the *F. graminearum* PH-1 strain, we employed a split marker strategy (75). This involved amplifying the left and right flanking regions of the target genes and combining them with a minimal gene cassette that carried the hygromycin phosphotransferase (HPH) gene under the control of the trpC promoter from *Aspergillus nidulans*. To achieve this, we conducted fusion PCR, following the previously described method (76), and the specific primers used for targeted gene deletion can be found in Table S3. In brief, we amplified the left and right flanking regions of the target genes separately, using L5 and L3 primer pairs and R5 and R3 primer pairs, respectively. The L3 and R5 primers contained 27-nucleotide (nt)-long overhang sequences that were complementary to the 5′ and 3′ ends of the minimal HPH cassette (1,376 bp in length). The HPH cassette was obtained from the pCB1004 plasmid (77), and amplified using HYG-F and HYG-R primers. Subsequently, we merged the PCR amplicons through overlap extension, assembling the left flanking region and the HPH cassette, or the right flanking region and the HPH cassette. The split marker constructs were obtained by amplifying the fused amplicons using nested primer pairs (N5 and HY-R primers for the left-half construct and YG-F and N3 primers for the right-half construct). Finally, we introduced the two split marker constructs into protoplasts through polyethylene glycol-mediated transformation (78). Following transformation, transformants resistant to 200 mg/mL of hygromycin were examined for replacement of the target gene with the HPH cassette by diagnostic PCR checks, in which L5 and R3 primers that aneal to flanking sequences of the homologous recombination event were used to confirm homologous integration of the HPH cassette into the target loci.

For the generation of MTA1-overexpressing strains, the coding sequence of MTA1 was cloned to pDS23 plasmid digested with BglII and HindIII restriction enzymes (79), using the In-Fusion HD Cloning kit (Takara Bio, Otsu, Japan). Five micrograms of the plasmid harboring MTA1 was transformed into protoplasts of the WT strain. Following transformation, transformants resistant to 200 mg/mL of nourseothricin (Jena Bioscience, Jena, Germany) were examined for introduction of an additional copy of MTA1 by diagnostic PCR check using primer pair, MTA1_OE_RT_fwd and MTA1_RT_rev. Primers used in this study are listed in Table S3.

### Sexual development and hyphal growth measurement

Carrot agar plates (60 mm in diameter) (80) were inoculated by placing an agar block containing hyphae of *F. graminearum* at the center. The plates were incubated at room temperature under constant light. Six days after incubation, mycelia were removed by gently scraping the surface with a spatula, and then 0.9 mL of 2.5% Tween 60 (Sigma-Aldrich, St. Louis, MO, USA) was applied to the surface to assist the formation of perithecia. Sexual development in knockout mutants was observed by a stereomicroscopy for size and number formed. Squash mounts of young developing perithecia in water were examined using a compound microscope to check the morphology and maturity of ascospores.

To obtain macroconidia of the WT and MTA1-overexpressing strains, small agar blocks containing each strain were inoculated and cultured in carboxymethylcellulose medium for 3 days at 20°C (81). Macroconidia were harvested by filtration through two layers of Miracloth. Concentrations of macroconidia were adjusted to 1 × 10^8^ spores per mL, and 40 µL of spore suspensions was inoculated into 100 mL of q-PDB. Cultures were shaken at 150 rpm at room temperature. Mycelia were collected by filtration through one layer of Miracloth and oven-dried at 55°C for 2 days.

### M^6^A quantification

The m^6^A RNA methylation level was assessed using the EpiQuikTM m6A RNA methylation quantitative kit (Epigentek, Farmingdale, NY, USA). Briefly, 200 ng of total RNA was added and bound with the antibody in the strip wells, followed by the process of washing, capturing, and detecting antibody. Finally, the signals were detected colorimetrically by reading the absorbance at 450 nm. m^6^A levels were eventually calculated based on a constructed standard curve.

### RT-PCR and quantitative RT-PCR analyses

Total RNA was extracted from fresh conidia, hyphae, and perithecial tissues ground in liquid nitrogen using TRIzol reagent (Thermo Fisher Scientific) according to the manufacturer’s instructions together with the following extraction steps: two phenol (pH 4.6)-chloroform-isoamyl alcohol (25:24:1) extraction steps followed by two chloroform extraction steps after the initial TRIzol-chloroform phase separation. RNA pellets were dissolved in 88 µL of nuclease-free water and subjected to genomic DNA digestion with DNase (Qiagen Inc.). RNA samples were then concentrated using RNA Clean & Concentrator (Zymo Research). For reverse transcription (RT)-PCR analysis of MTA1, 200 ng of RNAs was reverse transcribed and amplified using the OneStep RT-PCR kit (Qiagen Inc.). MTA1 and EF1α (FGRRES_08811) were amplified for 34 cycles in the experiment for Fig. 4C, or 30 cycles in the experiment for Fig. 5A, respectively. The annealing temperature was 62°C. The primer set for amplifying EF1α was designed to amplify flanking exons, including two introns to detect possible genomic DNA contamination. For semi-quantitative RT-PCR assays, first-strand cDNA synthesis was prepared from 200 ng of total RNA using the iScript cDNA Synthesis Kit (Bio-Rad), according to the manufacturer’s instructions. Real-time RT-PCR analyses were performed using the CFX96 Touch Real-Time PCR Detection System (Bio-Rad). RT-PCR mixtures were composed of 1.5 µL of each primer (10 µM), 5 µL of SYBR Green Supermix (Bio-Rad), and 2 µL of cDNA (100 ng/mL). The PCR conditions consisted of an initial denaturing step at 95°C for 3 minutes, a denaturation step at 95°C for 10 s, both annealing and extension steps at 65°C for 30 s for 40 cycles, and 65 to 95°C with a 0.5°C increment and each temperature for 5 s to obtain the melting curve. The quantification of the relative expression levels was performed with the comparative cycle threshold method normalization (82), in that the expression level of MTA1 was normalized against the reference gene EF1α. The averages of the three biological replicates and standard deviations of the relative expression values were presented (Fig. S4). Primers used in the expression analysis are listed in Table S3.

### RNA-seq and differential expression analyses

Total RNA samples were extracted from fresh conidia of the WT, MTA1-KD1, and MTA1-OE5 strains that have been harvested from carboxymethylcellulose medium 4 days after incubation at 20°C (81). Three separate experiments were performed to produce conidia, and the conidia samples from each experiment were used as replicates for RNA-Seq analysis. Two micrograms of total RNA was sent to Macrogen Inc. (Seoul, Korea) for cDNA library construction, using the TruSeq Stranded Total RNA Library Prep Gold Kit (Illumina, San Diego, CA, USA), and for sequencing on the HiSeq4000 platform (Illumina). Raw reads (paired-end, 100 bp) were further processed and filtered, using the TrimGalore (v0.6.6) (https://www.bioinformatics.babraham.ac.uk/projects/trim_galore/). Filtered reads were mapped to the genome sequence of F. graminearum (NCBI accession: GCA_000240135.3), using the HISAT2 program (v2.1.0). The gene annotation file used in this study was Ensembl annotation v.32 (83). Mapped reads on genomic features, such as exon and intron, were calculated, using the htseq-count program. Gene expression levels in reads per kilobase per million mapped reads (RPKM) values were computed and normalized by effective library size estimated by trimmed mean of M values, using the edgeR R package (v3.26.8). For differential expression analysis, only genes with CPM values greater than 1 in at least three samples were kept for further analyses (9,792 out of 16,001 gene loci). Then, DE genes showing greater than a fourfold difference at an FDR of 5% were identified between the WT, MTA1-KD1, and MTA1-OE5 strains, using the limma R package (v3.28.21).

### DATA accessions

The RNA-seq data generated in the present work have been deposited in NCBI’s Sequence Read Archive and are accessible through SRA accessions from SRX21157089 to SRX21157097, which belong to the BioProject (accession, PRJNA998561). RNA-seq data for investigating the expression level changes in m^6^A factors throughout the life cycle of *F. graminearum* can be found in NCBI’s Gene Expression Omnibus GSE109088 for the conidial germination stages (S1–S3) and GSE109094 for the sexual development (S4–S9).

## Data Availability

All data generated or analyzed during this study are included in this published article and its supplementary information files.

## SUPPLEMENTAL MATERIAL

Supplemental figures. msphere.00552-23-s0001.pdf Figures S2 to S7.

Fig. S1 msphere.00552-23-s0002.pdf Maximum likelihood trees of potential m^6^A factors in fungi.

Table S1 msphere.00552-23-s0003.xlsx Metadata for UniProt protein sequences related to MTA1, IME4, KAR4, WTAP, VIRN, YTH1, and ALKBHs.

Table S2 msphere.00552-23-s0004.docx Numbers of putative m^6^A erasers found in 27 selected fungi.

Table S3 msphere.00552-23-s0005.docx Primers.

ASM does not own the copyrights to Supplemental Material that may be linked to, or accessed through, an article. The authors have granted ASM a non-exclusive, world-wide license to publish the Supplemental Material files. Please contact the corresponding author directly for reuse.

## References

[1] Hu J, Cai J, Xu T, Kang H. 2022. Epitranscriptomic mRNA modifications governing plant stress responses: underlying mechanism and potential application. Plant Biotechnol J 20:2245–2257. doi:10.1111/pbi.13913 36002976 PMC9674322

[2] Manavella PA, Godoy Herz MA, Kornblihtt AR, Sorenson R, Sieburth LE, Nakaminami K, Seki M, Ding Y, Sun Q, Kang H, Ariel FD, Crespi M, Giudicatti AJ, Cai Q, Jin H, Feng X, Qi Y, Pikaard CS. 2023. Beyond transcription: compelling open questions in plant RNA biology. Plant Cell 35:1626–1653. doi:10.1093/plcell/koac346 36477566 PMC10226580

[3] Motorin Y, Helm M. 2011. RNA nucleotide methylation. WIREs RNA 2:611–631. doi:10.1002/wrna.79 21823225

[4] Saletore Y, Meyer K, Korlach J, Vilfan ID, Jaffrey S, Mason CE. 2012. The birth of the epitranscriptome: deciphering the function of RNA modifications. Genome Biol 13:175. doi:10.1186/gb-2012-13-10-175 23113984 PMC3491402

[5] Perry RP, Kelley DE. 1974. Existence of methylated messenger RNA in mouse L cells. Cell 1:37–42. doi:10.1016/0092-8674(74)90153-6

[6] Desrosiers R, Friderici K, Rottman F. 1974. Identification of methylated nucleosides in messenger RNA from novikoff hepatoma cells. Proc Natl Acad Sci U S A 71:3971–3975. doi:10.1073/pnas.71.10.3971 4372599 PMC434308

[7] Fu Y, Dominissini D, Rechavi G, He C. 2014. Gene expression regulation mediated through reversible m6A RNA methylation. Nat Rev Genet 15:293–306. doi:10.1038/nrg3724 24662220

[8] Wang S, Sun C, Li J, Zhang E, Ma Z, Xu W, Li H, Qiu M, Xu Y, Xia W, Xu L, Yin R. 2017. Roles of RNA methylation by means of N6-methyladenosine (m6A) in human cancers. Cancer Letters 408:112–120. doi:10.1016/j.canlet.2017.08.030 28867248

[9] Bodi Z, Button JD, Grierson D, Fray RG. 2010. Yeast targets for mRNA methylation. Nucleic Acids Res 38:5327–5335. doi:10.1093/nar/gkq266 20421205 PMC2938207

[10] Hsu PJ, Shi H, He C. 2017. Epitranscriptomic influences on development and disease. Genome Biol 18:197. doi:10.1186/s13059-017-1336-6 29061143 PMC5654102

[11] Cai J, Hu J, Amara U, Park SJ, Li Y, Jeong D, Lee I, Xu T, Kang H. 2023. Arabidopsis N6-methyladenosine methyltransferase FIONA1 regulates floral transition by affecting the splicing of FLC and the stability of floral activators SPL3 and SEP3. J Exp Bot 74:864–877. doi:10.1093/jxb/erac461 36416766

[12] Fabian M, Gao M, Zhang X-N, Shi J, Vrydagh L, Kim S-H, Patel P, Hu AR, Lu H. 2023. The flowering time regulator FLK controls pathogen defense in Arabidopsis thaliana. Plant Physiol 191:2461–2474. doi:10.1093/plphys/kiad021 36662556 PMC10069895

[13] Amara U, Hu J, Cai J, Kang H. 2023. FLK is an mRNA m6A reader that regulates floral transition by modulating the stability and splicing of FLC in arabidopsis. Mol Plant 16:919–929. doi:10.1016/j.molp.2023.04.005 37050878

[14] Hu J, Manduzio S, Kang H. 2019. Epitranscriptomic RNA methylation in plant development and abiotic stress responses. Front Plant Sci 10:500. doi:10.3389/fpls.2019.00500 31110512 PMC6499213

[15] Jeon J, Lee SH. 2021. RNA modification and its implication in plant pathogenic fungi. Plant Pathol J 37:505–511. doi:10.5423/PPJ.RW.07.2021.0111 34897243 PMC8666238

[16] Yang Y, Hsu PJ, Chen Y-S, Yang Y-G. 2018. Dynamic transcriptomic m6A decoration: writers, erasers, readers and functions in RNA metabolism. Cell Res 28:616–624. doi:10.1038/s41422-018-0040-8 29789545 PMC5993786

[17] Zaccara S, Ries RJ, Jaffrey SR. 2019. Reading, writing and erasing mRNA methylation. Nat Rev Mol Cell Biol 20:608–624. doi:10.1038/s41580-019-0168-5 31520073

[18] Bokar JA, Shambaugh ME, Polayes D, Matera AG, Rottman FM. 1997. Purification and cDNA cloning of the AdoMet-binding subunit of the human mRNA (N6-adenosine)-methyltransferase. RNA 3:1233–1247. 9409616 PMC1369564

[19] Liu J, Yue Y, Han D, Wang X, Fu Y, Zhang L, Jia G, Yu M, Lu Z, Deng X, Dai Q, Chen W, He C. 2014. A METTL3–METTL14 complex mediates mammalian nuclear RNA N6-adenosine methylation. Nat Chem Biol 10:93–95. doi:10.1038/nchembio.1432 24316715 PMC3911877

[20] Ping X-L, Sun B-F, Wang L, Xiao W, Yang X, Wang W-J, Adhikari S, Shi Y, Lv Y, Chen Y-S, Zhao X, Li A, Yang Y, Dahal U, Lou X-M, Liu X, Huang J, Yuan W-P, Zhu X-F, Cheng T, Zhao Y-L, Wang X, Rendtlew Danielsen JM, Liu F, Yang Y-G. 2014. Mammalian WTAP is a regulatory subunit of the RNA N6-methyladenosine methyltransferase. Cell Res 24:177–189. doi:10.1038/cr.2014.3 24407421 PMC3915904

[21] Horiuchi K, Kawamura T, Iwanari H, Ohashi R, Naito M, Kodama T, Hamakubo T. 2013. Identification of Wilms’ tumor 1-associating protein complex and its role in alternative splicing and the cell cycle. J Biol Chem 288:33292–33302. doi:10.1074/jbc.M113.500397 24100041 PMC3829175

[22] Yue Y, Liu J, Cui X, Cao J, Luo G, Zhang Z, Cheng T, Gao M, Shu X, Ma H, Wang F, Wang X, Shen B, Wang Y, Feng X, He C, Liu J. 2018. VIRMA mediates preferential M6A mRNA methylation in 3′UTR and near stop codon and associates with alternative polyadenylation. Cell Discov 4:10. doi:10.1038/s41421-018-0019-0 29507755 PMC5826926

[23] Zhang Z, Theler D, Kaminska KH, Hiller M, de la Grange P, Pudimat R, Rafalska I, Heinrich B, Bujnicki JM, Allain F-T, Stamm S. 2010. The YTH domain is a novel RNA binding domain. J Biol Chem 285:14701–14710. doi:10.1074/jbc.M110.104711 20167602 PMC2863249

[24] Du H, Zhao Y, He J, Zhang Y, Xi H, Liu M, Ma J, Wu L. 2016. YTHDF2 destabilizes m6A-containing RNA through direct recruitment of the CCR4-NOT deadenylase complex. Nat Commun 7:12626. doi:10.1038/ncomms12626 27558897 PMC5007331

[25] Wang X, Lu Z, Gomez A, Hon GC, Yue Y, Han D, Fu Y, Parisien M, Dai Q, Jia G, Ren B, Pan T, He C. 2014. N6-methyladenosine-dependent regulation of messenger RNA stability. Nature 505:117–120. doi:10.1038/nature12730 24284625 PMC3877715

[26] Zheng G, Dahl JA, Niu Y, Fedorcsak P, Huang C-M, Li CJ, Vågbø CB, Shi Y, Wang W-L, Song S-H, et al.. 2013. ALKBH5 is a mammalian RNA demethylase that impacts RNA metabolism and mouse fertility. Mol Cell 49:18–29. doi:10.1016/j.molcel.2012.10.015 23177736 PMC3646334

[27] Jia G, Fu Y, Zhao X, Dai Q, Zheng G, Yang Y, Yi C, Lindahl T, Pan T, Yang Y-G, He C. 2011. N6-Methyladenosine in nuclear RNA is a major substrate of the obesity-associated FTO. Nat Chem Biol 7:885–887. doi:10.1038/nchembio.687 22002720 PMC3218240

[28] Yadav PK, Rajasekharan R. 2017. The m6A methyltransferase Ime4 epitranscriptionally regulates triacylglycerol metabolism and vacuolar morphology in haploid yeast cells. J Biol Chem 292:13727–13744. doi:10.1074/jbc.M117.783761 28655762 PMC5566527

[29] Clancy MJ, Shambaugh ME, Timpte CS, Bokar JA. 2002. Induction of sporulation in Saccharomyces cerevisiae leads to the formation of N6-methyladenosine in mRNA: a potential mechanism for the activity of the IME4 gene. Nucleic Acids Res 30:4509–4518. doi:10.1093/nar/gkf573 12384598 PMC137137

[30] Yadav PK, Rajasekharan R. 2018. The m6A methyltransferase Ime4 and mitochondrial functions in yeast. Curr Genet 64:353–357. doi:10.1007/s00294-017-0758-8 28975387

[31] Agarwala SD, Blitzblau HG, Hochwagen A, Fink GR. 2012. RNA methylation by the MIS complex regulates a cell fate decision in yeast. PLoS Genet 8:e1002732. doi:10.1371/journal.pgen.1002732 22685417 PMC3369947

[32] Park ZM, Belnap E, Remillard M, Rose MD. 2023. Vir1p, the yeast homolog of virilizer, is required for mRNA m6A methylation and meiosis. Genetics 224:iyad043. doi:10.1093/genetics/iyad043 36930734 PMC10474941

[33] Ensinck I, Maman A, Albihlal WS, Lassandro M, Salzano G, Sideri T, Howell SA, Calvani E, Patel H, Bushkin G, Ralser M, Snijders AP, Skehel M, Casañal A, Schwartz S, van Werven FJ. 2023. The yeast RNA methylation complex consists of conserved yet reconfigured components with m6A-dependent and independent roles. Elife 12:RP87860. doi:10.7554/eLife.87860 37490041 PMC10393049

[34] Kang H-J, Jeong S-J, Kim K-N, Baek I-J, Chang M, Kang C-M, Park Y-S, Yun C-W. 2014. A novel protein, Pho92, has a conserved YTH domain and regulates phosphate metabolism by decreasing the mRNA stability of PHO4 in Saccharomyces cerevisiae. Biochem J 457:391–400. doi:10.1042/BJ20130862 24206186

[35] Schwartz S, Agarwala SD, Mumbach MR, Jovanovic M, Mertins P, Shishkin A, Tabach Y, Mikkelsen TS, Satija R, Ruvkun G, Carr SA, Lander ES, Fink GR, Regev A. 2013. High-resolution mapping reveals a conserved, widespread, dynamic mRNA methylation program in yeast meiosis. Cell 155:1409–1421. doi:10.1016/j.cell.2013.10.047 24269006 PMC3956118

[36] Varier RA, Sideri T, Capitanchik C, Manova Z, Calvani E, Rossi A, Edupuganti RR, Ensinck I, Chan VWC, Patel H, Kirkpatrick J, Faull P, Snijders AP, Vermeulen M, Ralser M, Ule J, Luscombe NM, van Werven FJ. 2022. N6-methyladenosine (m6A) reader Pho92 is recruited co-transcriptionally and couples translation to mRNA decay to promote meiotic fitness in yeast. Elife 11:e84034. doi:10.7554/eLife.84034 36422864 PMC9731578

[37] Scutenaire J, Plassard D, Matelot M, Villa T, Zumsteg J, Libri D, Séraphin B. 2023. The S. cerevisiae m6A-reader Pho92 promotes timely meiotic recombination by controlling key methylated transcripts. Nucleic Acids Res 51:517–535. doi:10.1093/nar/gkac640 35934316 PMC9881176

[38] Harigaya Y, Tanaka H, Yamanaka S, Tanaka K, Watanabe Y, Tsutsumi C, Chikashige Y, Hiraoka Y, Yamashita A, Yamamoto M. 2006. Selective elimination of messenger RNA prevents an incidence of untimely meiosis. Nature 442:45–50. doi:10.1038/nature04881 16823445

[39] Shichino Y, Otsubo Y, Kimori Y, Yamamoto M, Yamashita A. 2018. YTH-RNA-binding protein prevents deleterious expression of meiotic proteins by tethering their mRNAs to nuclear foci. Elife 7:e32155. doi:10.7554/eLife.32155 29424342 PMC5807050

[40] Trail F. 2009. For blighted waves of grain: Fusarium graminearum in the postgenomics era. Plant Physiol 149:103–110. doi:10.1104/pp.108.129684 19126701 PMC2613717

[41] Wang Z, Kim W, Wang Y-W, Yakubovich E, Dong C, Trail F, Townsend JP, Yarden O. 2023. The sordariomycetes: an expanding resource with big data for mining in evolutionary genomics and transcriptomics. Front Fungal Biol 4:1214537. doi:10.3389/ffunb.2023.1214537 37746130 PMC10512317

[42] Ma L-J, Geiser DM, Proctor RH, Rooney AP, O’Donnell K, Trail F, Gardiner DM, Manners JM, Kazan K. 2013. Fusarium pathogenomics. Annu Rev Microbiol 67:399–416. doi:10.1146/annurev-micro-092412-155650 24024636

[43] Feng C, Cao X, Du Y, Chen Y, Xin K, Zou J, Jin Q, Xu J-R, Liu H, Lin X. 2022. Uncovering cis-regulatory elements important for A-to-I RNA editing in Fusarium graminearum. mBio 13:e0187222. doi:10.1128/mbio.01872-22 36102513 PMC9600606

[44] Xin K, Zhang Y, Fan L, Qi Z, Feng C, Wang Q, Jiang C, Xu J-R, Liu H. 2023. Experimental evidence for the functional importance and adaptive advantage of A-to-I RNA editing in fungi. Proc Natl Acad Sci U S A 120:e2219029120. doi:10.1073/pnas.2219029120 36917661 PMC10041177

[45] Cao S, He Y, Hao C, Xu Y, Zhang H, Wang C, Liu H, Xu J-R. 2017. RNA editing of the AMD1 gene is important for ascus maturation and ascospore discharge in Fusarium graminearum. Sci Rep 7:4617. doi:10.1038/s41598-017-04960-7 28676631 PMC5496914

[46] Shi Y, Wang H, Wang J, Liu X, Lin F, Lu J. 2019. N6-methyladenosine RNA methylation is involved in virulence of the rice blast fungus Pyricularia oryzae (syn magnaporthe oryzae). FEMS Microbiol Lett 366. doi:10.1093/femsle/fny286 30535195

[47] Ren Z, Tang B, Xing J, Liu C, Cai X, Hendy A, Kamran M, Liu H, Zheng L, Huang J, Chen X-L. 2022. MTA1‐mediated RNA m 6 A modification regulates autophagy and is required for infection of the rice blast fungus. New Phytol 235:247–262. doi:10.1111/nph.18117 35338654

[48] Duan H-C, Wei L-H, Zhang C, Wang Y, Chen L, Lu Z, Chen PR, He C, Jia G. 2017. ALKBH10B is an RNA N 6-methyladenosine demethylase affecting arabidopsis floral transition. Plant Cell 29:2995–3011. 29180595 10.1105/tpc.16.00912 PMC5757257

[49] Amara U, Shoaib Y, Kang H. 2022. ALKBH9C, a potential RNA m6A demethylase, regulates the response of arabidopsis to abiotic stresses and abscisic acid. Plant Cell Environ 45:3566–3581. doi:10.1111/pce.14447 36148771

[50] Shoaib Y, Hu J, Manduzio S, Kang H. 2021. Alpha-ketoglutarate-dependent dioxygenase homolog 10B, an N6-methyladenosine mRNA demethylase, plays a role in salt stress and abscisic acid responses in Arabidopsis thaliana. Physiol Plant 173:1078–1089. doi:10.1111/ppl.13505 34309025

[51] Meza TJ, Moen MN, Vågbø CB, Krokan HE, Klungland A, Grini PE, Falnes PØ. 2012. The DNA dioxygenase ALKBH2 protects Arabidopsis thaliana against methylation damage. Nucleic Acids Res 40:6620–6631. doi:10.1093/nar/gks327 22532610 PMC3413135

[52] Liefke R, Windhof-Jaidhauser IM, Gaedcke J, Salinas-Riester G, Wu F, Ghadimi M, Dango S. 2015. The oxidative demethylase ALKBH3 marks hyperactive gene promoters in human cancer cells. Genome Med 7:66. doi:10.1186/s13073-015-0180-0 26221185 PMC4517488

[53] Zhao S, Devega R, Francois A, Kidane D. 2021. Human ALKBH6 is required for maintenance of genomic stability and promoting cell survival during exposure of alkylating agents in pancreatic cancer. Front Genet 12:635808. doi:10.3389/fgene.2021.635808 33897761 PMC8058185

[54] Ma L, Lu H, Tian Z, Yang M, Ma J, Shang G, Liu Y, Xie M, Wang G, Wu W, Zhang Z, Dai S, Chen Z. 2022. Structural insights into the interactions and epigenetic functions of human nucleic acid repair protein ALKBH6. J Biol Chem 298:101671. doi:10.1016/j.jbc.2022.101671 35120926 PMC8892091

[55] Westbye MP, Feyzi E, Aas PA, Vågbø CB, Talstad VA, Kavli B, Hagen L, Sundheim O, Akbari M, Liabakk N-B, Slupphaug G, Otterlei M, Krokan HE. 2008. Human AlkB homolog 1 is a mitochondrial protein that demethylates 3-methylcytosine in DNA and RNA. J Biol Chem 283:25046–25056. doi:10.1074/jbc.M803776200 18603530 PMC3259822

[56] Ougland R, Lando D, Jonson I, Dahl JA, Moen MN, Nordstrand LM, Rognes T, Lee JT, Klungland A, Kouzarides T, Larsen E. 2012. ALKBH1 is a histone H2A dioxygenase involved in neural differentiation. Stem Cells 30:2672–2682. doi:10.1002/stem.1228 22961808 PMC3546389

[57] Liu F, Clark W, Luo G, Wang X, Fu Y, Wei J, Wang X, Hao Z, Dai Q, Zheng G, Ma H, Han D, Evans M, Klungland A, Pan T, He C. 2016. ALKBH1-mediated tRNA demethylation regulates translation. Cell 167:1897. doi:10.1016/j.cell.2016.11.045 27984735

[58] Zhang C, Samanta D, Lu H, Bullen JW, Zhang H, Chen I, He X, Semenza GL. 2016. Hypoxia induces the breast cancer stem cell phenotype by HIF-dependent and ALKBH5-mediated m6A-demethylation of NANOG mRNA. Proc Natl Acad Sci U S A 113:E2047–E2056. doi:10.1073/pnas.1602883113 27001847 PMC4833258

[59] Zhang S, Zhao BS, Zhou A, Lin K, Zheng S, Lu Z, Chen Y, Sulman EP, Xie K, Bögler O, Majumder S, He C, Huang S. 2017. m6A demethylase ALKBH5 maintains tumorigenicity of glioblastoma stem-like cells by sustaining FOXM1 expression and cell proliferation program. Cancer Cell 31:591–606. doi:10.1016/j.ccell.2017.02.013 28344040 PMC5427719

[60] Martínez-Pérez M, Aparicio F, López-Gresa MP, Bellés JM, Sánchez-Navarro JA, Pallás V. 2017. Arabidopsis m6A demethylase activity modulates viral infection of a plant virus and the m6A abundance in its genomic RNAs. Proc Natl Acad Sci U S A 114:10755–10760. 28923956 10.1073/pnas.1703139114 PMC5635872

[61] Miguel-Rojas C, Cavinder B, Townsend JP, Trail F. 2023. Comparative transcriptomics of Fusarium graminearum and Magnaporthe oryzae spore germination leading up to infection. mBio 14:e0244222. doi:10.1128/mbio.02442-22 36598191 PMC9973345

[62] Kim W, Miguel-Rojas C, Wang J, Townsend JP, Trail F. 2018. Developmental dynamics of long noncoding RNA expression during sexual fruiting body formation in Fusarium graminearum. mBio 9:e01292-18. doi:10.1128/mBio.01292-18 30108170 PMC6094484

[63] Park ZM, Sporer A, Kraft K, Lum K, Blackman E, Belnap E, Yellman C, Rose MD. 2023. Kar4, the yeast homolog of METTL14, is required for mRNA m6A methylation and meiosis. bioRxiv:2023.01.29.526094. doi:10.1101/2023.01.29.526094 PMC10470960 37603553

[64] Kim W, Wang Z, Kim H, Pham K, Tu Y, Townsend JP, Trail F. 2022. Transcriptional divergence underpinning sexual development in the fungal class sordariomycetes. mBio 13:e0110022. doi:10.1128/mbio.01100-22 35638737 PMC9239162

[65] Liu H, Li Y, Chen D, Qi Z, Wang Q, Wang J, Jiang C, Xu J-R. 2017. A-to-I RNA editing is developmentally regulated and generally adaptive for sexual reproduction in Neurospora crassa. Proc Natl Acad Sci U S A 114:E7756–E7765. doi:10.1073/pnas.1702591114 28847945 PMC5604002

[66] Wang C, Xu J-R, Liu H. 2016. A-to-I RNA editing independent of ADARs in filamentous fungi. RNA Biol 13:940–945. doi:10.1080/15476286.2016.1215796 27533598 PMC5056780

[67] Teichert I, Dahlmann TA, Kück U, Nowrousian M. 2017. RNA editing during sexual development occurs in distantly related filamentous ascomycetes. Genome Biol Evol 9:855–868. doi:10.1093/gbe/evx052 28338982 PMC5381528

[68] Amses KR, Simmons DR, Longcore JE, Mondo SJ, Seto K, Jerônimo GH, Bonds AE, Quandt CA, Davis WJ, Chang Y, et al.. 2022. Diploid-dominant life cycles characterize the early evolution of fungi. Proc Natl Acad Sci U S A 119:e2116841119. doi:10.1073/pnas.2116841119 36037379 PMC9457484

[69] Merényi Z, Krizsán K, Sahu N, Liu X-B, Bálint B, Stajich JE, Spatafora JW, Nagy LG. 2023. Genomes of fungi and relatives reveal delayed loss of ancestral gene families and evolution of key fungal traits. Nat Ecol Evol 7:1221–1231. doi:10.1038/s41559-023-02095-9 37349567 PMC10406608

[70] van Kempen M, Kim SS, Tumescheit C, Mirdita M, Lee J, Gilchrist CLM, Söding J, Steinegger M. 2023. Fast and accurate protein structure search with foldseek. Nat Biotechnol. doi:10.1038/s41587-023-01773-0 PMC10869269 37156916

[71] Ruperti F, Papadopoulos N, Musser JM, Mirdita M, Steinegger M, Arendt D. 2023. Cross-phyla protein annotation by structural prediction and alignment. Genome Biol 24:113. doi:10.1186/s13059-023-02942-9 37173746 PMC10176882

[72] Katoh K, Standley DM. 2013. MAFFT multiple sequence alignment software version 7: improvements in performance and usability. Mol Biol Evol 30:772–780. doi:10.1093/molbev/mst010 23329690 PMC3603318

[73] Capella-Gutiérrez S, Silla-Martínez JM, Gabaldón T. 2009. trimAl: a tool for automated alignment trimming in large-scale phylogenetic analyses. Bioinformatics 25:1972–1973. doi:10.1093/bioinformatics/btp348 19505945 PMC2712344

[74] Stamatakis A. 2014. RAxML version 8: a tool for phylogenetic analysis and post-analysis of large phylogenies. Bioinformatics 30:1312–1313. doi:10.1093/bioinformatics/btu033 24451623 PMC3998144

[75] Catlett NL, Lee B-N, Yoder OC, Turgeon BG. 2003. Split-marker recombination for efficient targeted deletion of fungal genes. Fungal Genet Newsl 50:9–11. doi:10.4148/1941-4765.1150

[76] Yu J-H, Hamari Z, Han K-H, Seo J-A, Reyes-Domínguez Y, Scazzocchio C. 2004. Double-joint PCR: a PCR-based molecular tool for gene manipulations in filamentous fungi. Fungal Genet Biol 41:973–981. doi:10.1016/j.fgb.2004.08.001 15465386

[77] Carroll AM, Sweigard JA, Valent B. 1994. Improved vectors for selecting resistance to hygromycin. Fungal Genet Newsl 41:22. doi:10.4148/1941-4765.1367

[78] Hallen-Adams HE, Cavinder BL, Trail F. 2011. *Fusarium graminearum* from expression analysis to functional assays, p 79–101. In Xu JR, BH Bluhm (ed), Fungal genomics: methods in molecular biology. Humana Press, Totowa, NJ. 10.1007/978-1-61779-040-9_6 21590414

[79] Teichert I, Wolff G, Kück U, Nowrousian M. 2012. Combining laser microdissection and RNA-seq to chart the transcriptional landscape of fungal development. BMC Genomics 13:511. doi:10.1186/1471-2164-13-511 23016559 PMC3472292

[80] Klittich CJR, Leslie JF. 1988. Nitrate reduction mutants of Fusarium moniliforme (Gibberella fujikuroi). Genetics 118:417–423. doi:10.1093/genetics/118.3.417 17246415 PMC1203296

[81] Cappellini RA, Peterson JL. 1965. Macroconidium formation in submerged cultures by a non-sporulating strain of Gibberella zeae. Mycologia 57:962–966. doi:10.1080/00275514.1965.12018285

[82] Schmittgen TD, Livak KJ. 2008. Analyzing real-time PCR data by the comparative CT method. Nat Protoc 3:1101–1108. doi:10.1038/nprot.2008.73 18546601

[83] King R, Urban M, Hammond-Kosack MCU, Hassani-Pak K, Hammond-Kosack KE. 2015. The completed genome sequence of the pathogenic ascomycete fungus Fusarium graminearum. BMC Genomics 16:544. doi:10.1186/s12864-015-1756-1 26198851 PMC4511438

