# Supplementary material for: Phylogenetic and functional analyses of N6-methyladenosine RNA methylation factors in the wheat scab fungus Fusarium graminearum
Source: mSphere. 2023 Dec 12;9(1):e00552-23. doi: 10.1128/msphere.00552-23 (PMC10826363; doi:10.1128/msphere.00552-23)
Supplement: Supplemental figures. — Figures S2 to S7. [file msphere.00552-23-s0001.pdf]

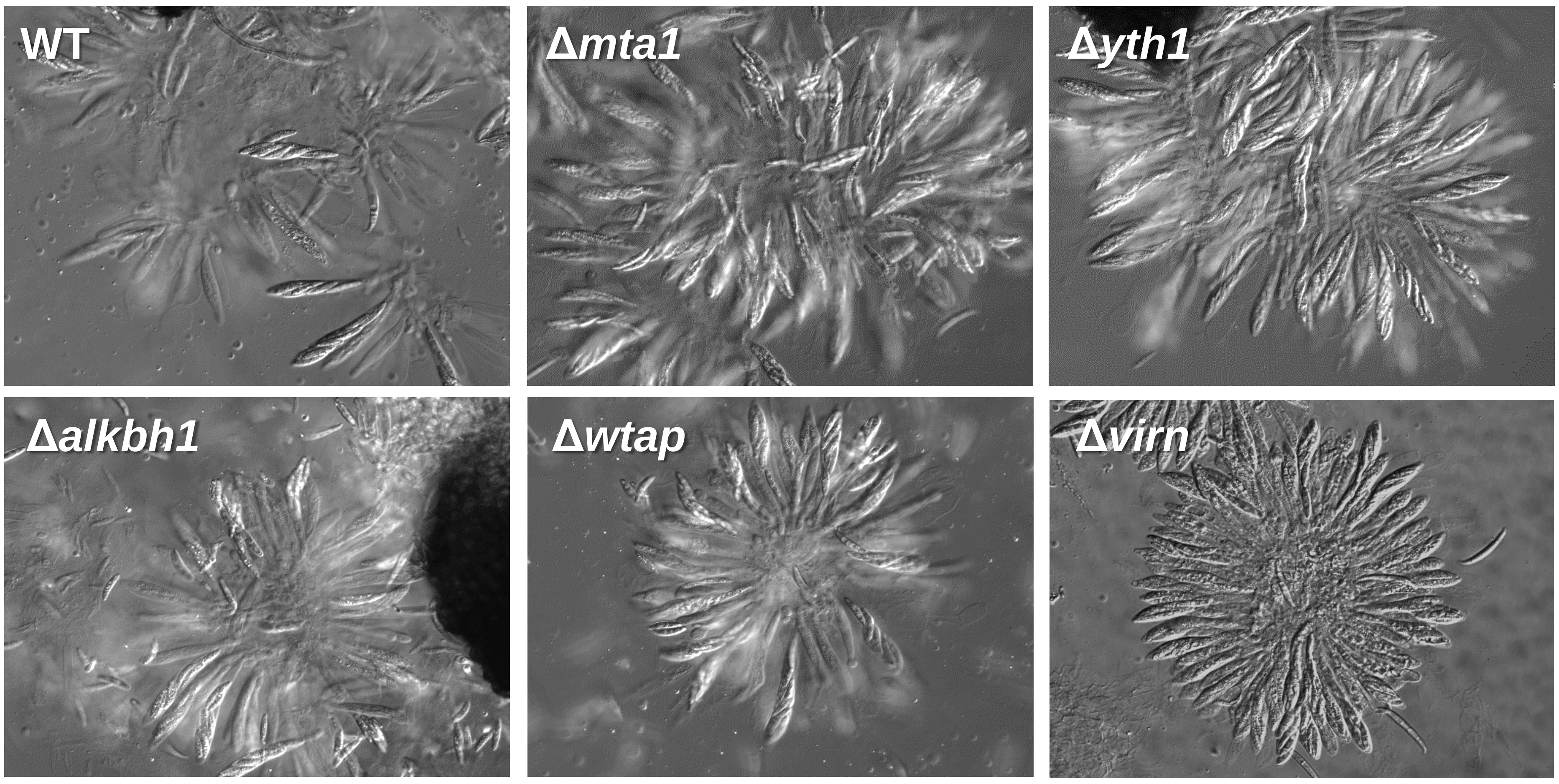

Supplementary Fig. S2. Normal ascospore production in m^{6}A factor mutants. Squash mounts of perithecia were observed with a compound microscope (400× magnification). Mature ascospores were observed in the WT and deletion mutants lacking m^{6}A factors. Photographs were taken 6 days after induction of sexual development.

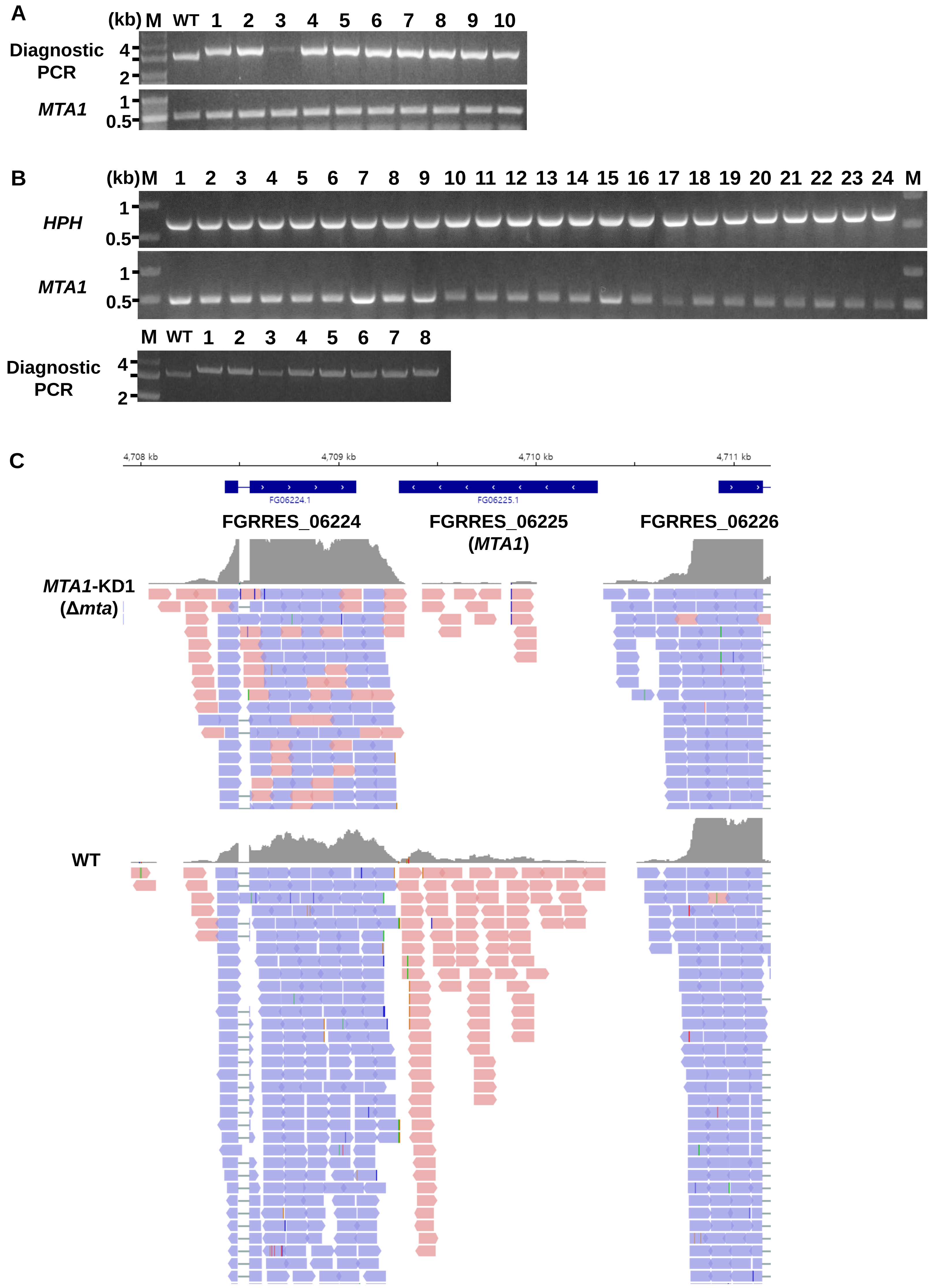

Supplementary Fig. S3. Genotyping for *MTA1*-deletion mutants. (**A**) Diagnostic PCRs confirmed homologous integration of the split marker constructs to the target gene loci (upper panel). Note the PCR band size difference due to the gene replacement between the wild-type (WT) and Δ*mta* strains obtained from the second round of single spore isolation (1–10). Amplification of *MTA1* in the WT and Δ*mta* strains (lower panel). (**B**) Amplification of the hygromycin phosphotransferase gene cassette (*HPH*) in the WT and newly generated Δ*mta* strains (1–24) (upper panel) and amplification of *MTA1* in the WT and newly generated Δ*mta* strains (1–24) (middle panel). Diagnostic PCRs confirmed homologous integration of the split marker constructs to the target gene loci in selected Δ*mta* strains (1–8) (lower panel). (**C**) Images of Integrative Genomics Viewer for the *MTA1* locus in the WT and *MTA*1-KD1 (Δ*mta*) strains. Mapped reads from RNA-seq data from three biological replicates were pooled and visualized. Total mapped reads for the WT and *MTA*1-KD1 were 68,583,046 and 81,845,383, respectively. Note that there were still mapped reads on the *MTA1* locus in the *MTA*1-KD1 (Δ*mta*) strain.

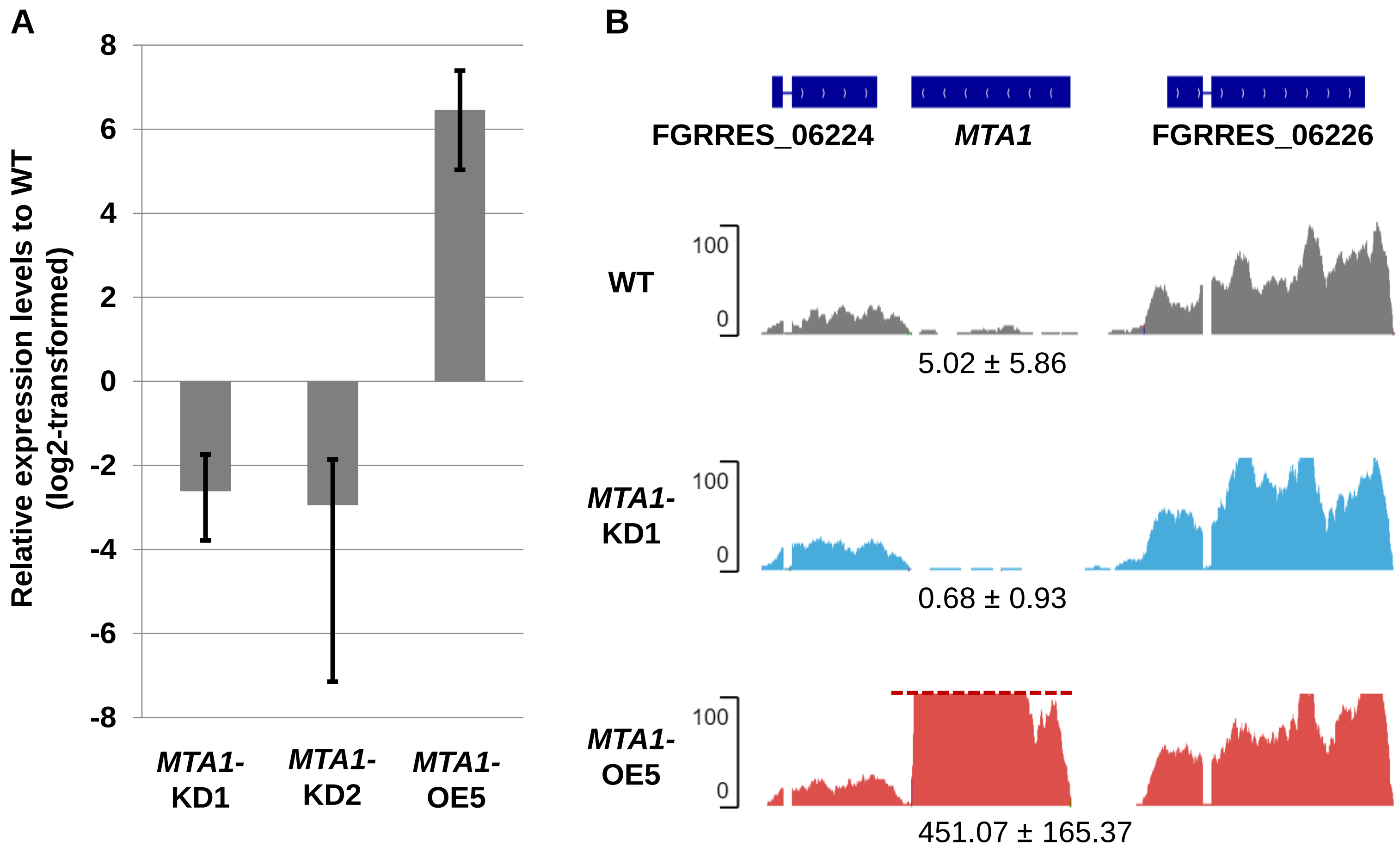

Supplementary Fig. S4. *MTA1* overexpression and knockdown. (**A**) Relative expression levels of *MTA1* in the *MTA*1-KD1 and *MTA*1-OE5 strains were estimated by semi-quantitative RT-PCR. The expression levels of *MTA1* were normalized against the reference gene, *EF1α*. (**B**) Visualization of transcriptome data on the *MTA1* locus. Mapped reads of three biological replicates were pooled, then subsampled to 20 million reads for visual comparison of expression levels between the wild-type (WT), *MTA*1-KD1 and *MTA*1-OE5 strains. Numbers on the *MTA1* locus indicate averaged RPKM values ± standard deviation.

Supplementary Fig. S5

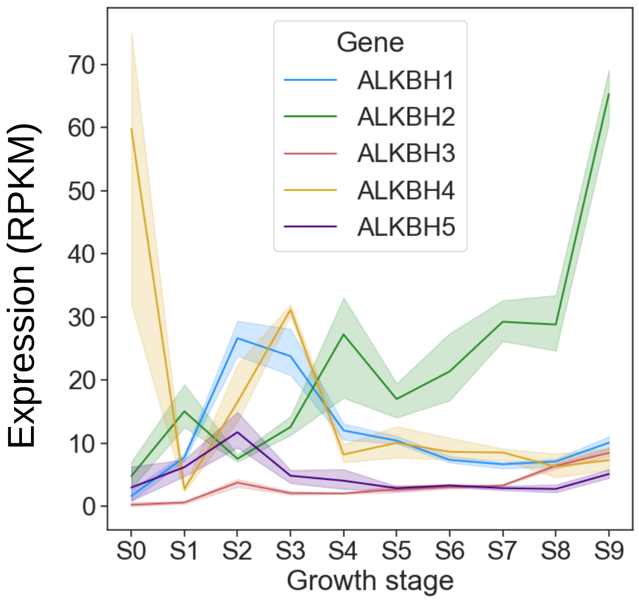

Growth Stages of *F. graminearum*

S0 – conidia harvested from CMC medium
S1 – 15 min after incubation on Bird medium
S2 – 3 h after incubation (polar growth)
S3 – 11 h after incubation (hyphal branching)
S4 – 2 h after sexual induction on Carrot medium
S5 – 24 h after sexual induction (ascogonia)
S6 – 48 h after sexual induction (perithecial wall)
S7 – 72 h after sexual induction (paraphyses)
S8 – 96 h after sexual induction (asci)
S9 – 144 h after sexual induction (ascospores)

Supplementary Fig. S5. Dynamic expression patterns of potential m^6A eraser genes. Average values for RPKM values for three biological replicates samples were plotted. Bands surrounding the line plots indicate 95% confidence intervals of the means. The *x*-axis are different growth stages of *F. graminearum* (S0–S9). See the right box for the description of vegetative and sexual growth stages. Gene ID for potential m^6A erasers are ALKBH1 (FGRRES_16652), ALKBH2 (FGRRES_01255), ALKBH3 (FGRRES_16456), ALKBH4 (FGRRES_09872) and ALKBH5 (FGRRES_20373).

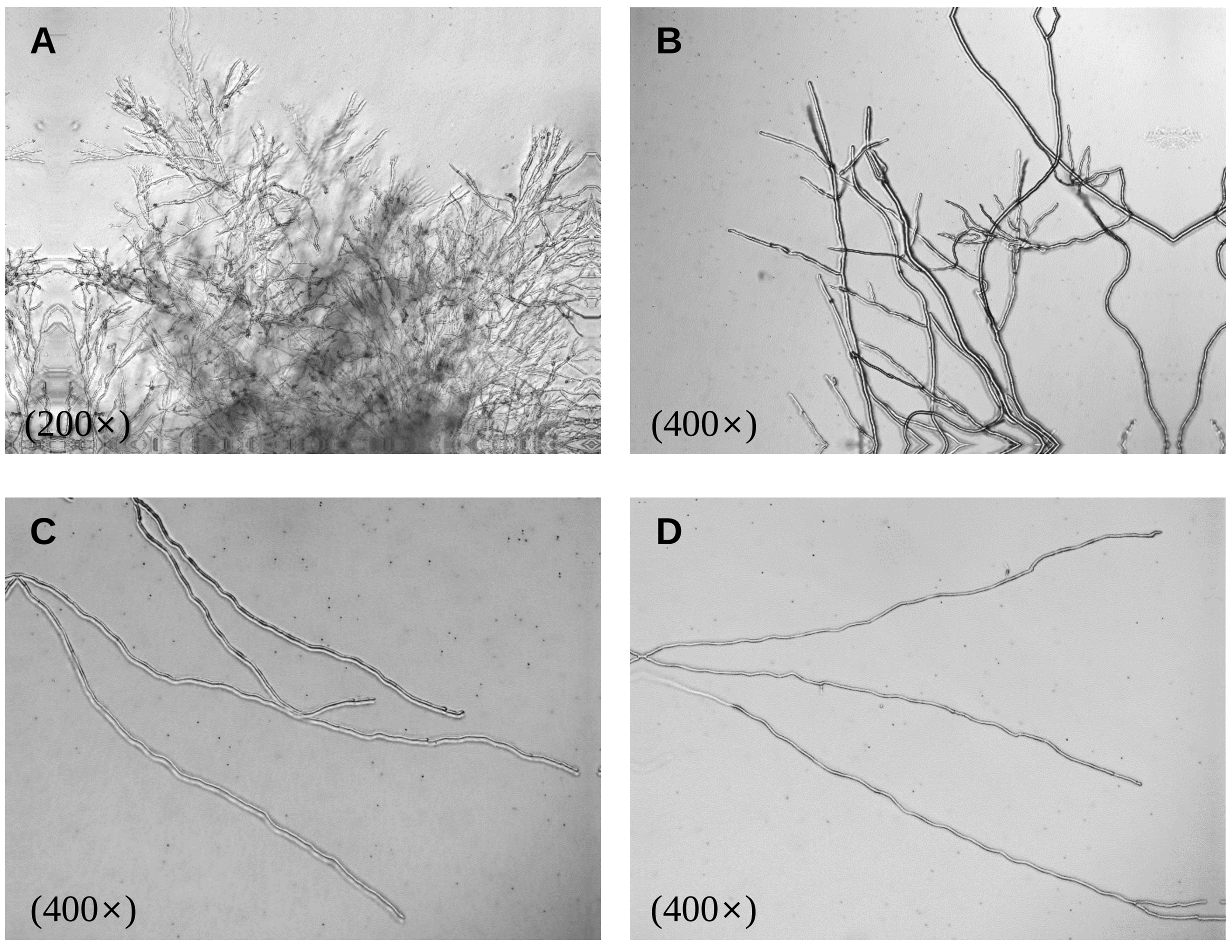

Supplementary Fig. S6. Reduced hyphal branching in the *MTA*1-OE5 strain. Actively growing hyphae of the wild-type strain (**A** and **B**) and *MTA*1-OE5 strain (**C** and **D**) on potato dextrose agar medium.

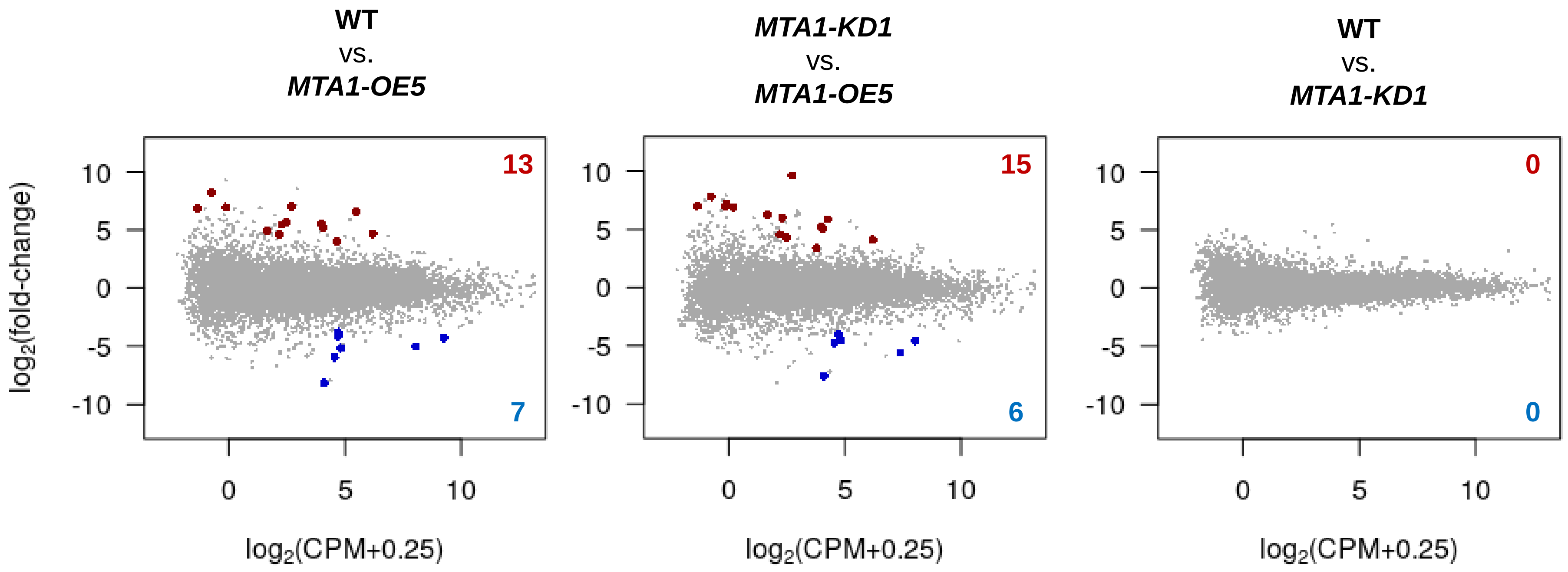

Supplementary Fig. S7. MA plots for differential expression analyses. Scatterplots of log2-transformed fold-change versus log2-transformed CPM (counts per million mapped reads) were displayed for 9,792 genes in comparisons between the wild-type (WT), *MTA*1-KD1 and *MTA*1-OE5 strains . Up-regulated genes in the *MTA*1-OE5 strain are highlighted red, while down-regulated genes are highlighted in blue. The numbers of up- and down-regulated genes were shown in each plot.
