# Supplementary material for: Phylogenetic and functional analyses of N6-methyladenosine RNA methylation factors in the wheat scab fungus Fusarium graminearum
Source: mSphere. 2023 Dec 12;9(1):e00552-23. doi: 10.1128/msphere.00552-23 (PMC10826363; doi:10.1128/msphere.00552-23)

Supplmentary Fig. 1A. A maximum likelihood tree of IME4 in fungi

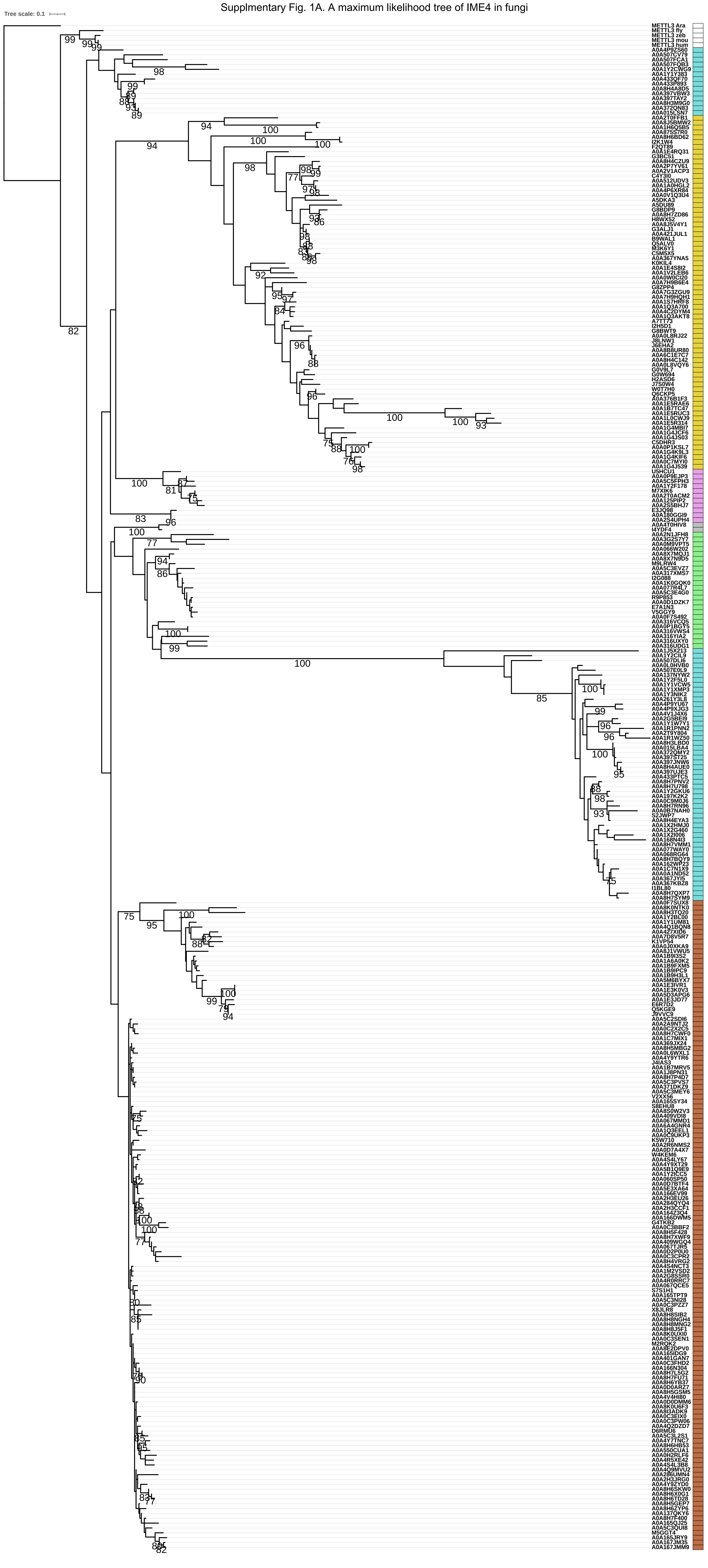

Supplementary Fig. 1B. A maximum likelihood tree of KAR4 in fungi

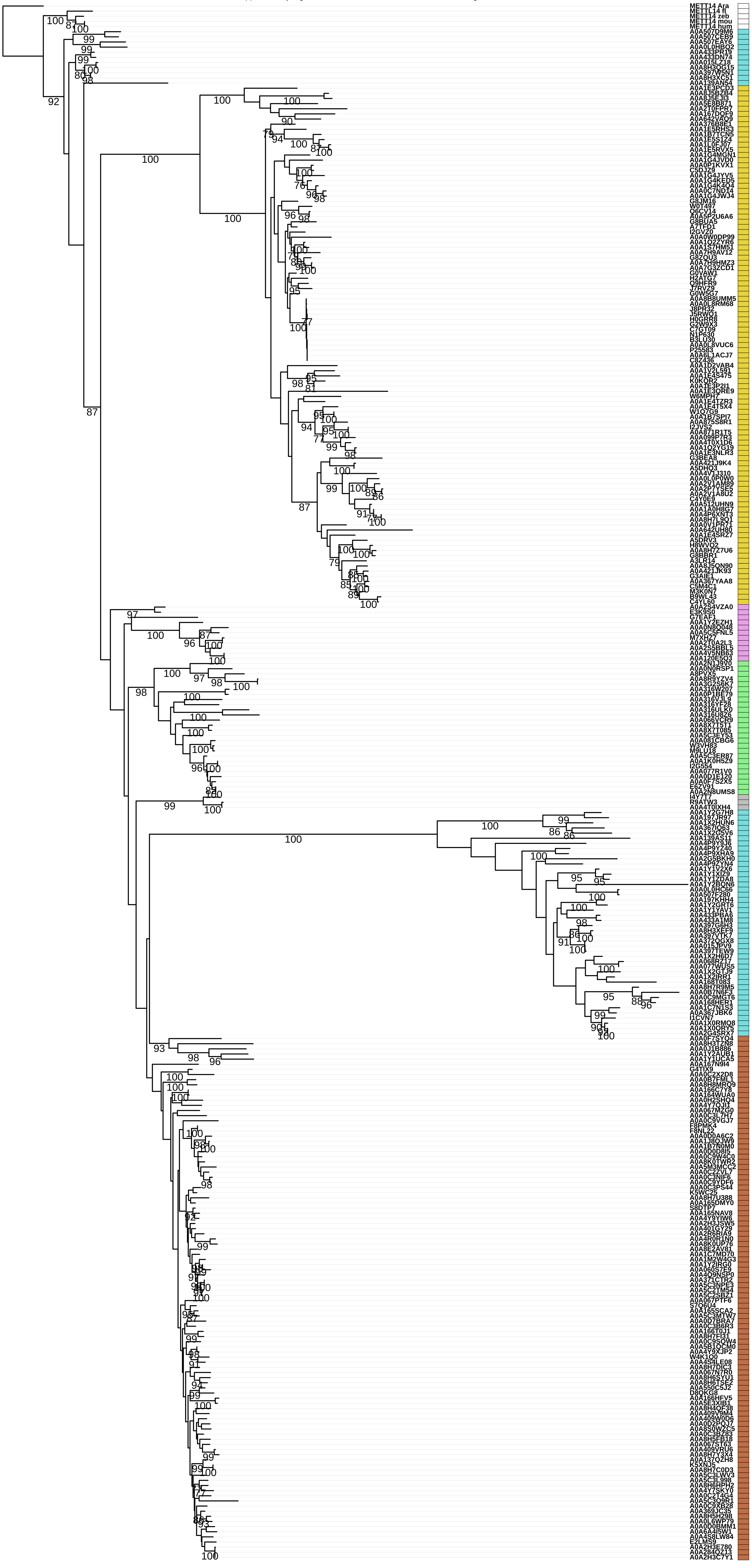

Supplementary Fig. 1C. A maximum likelihood tree of MTA1 in fungi

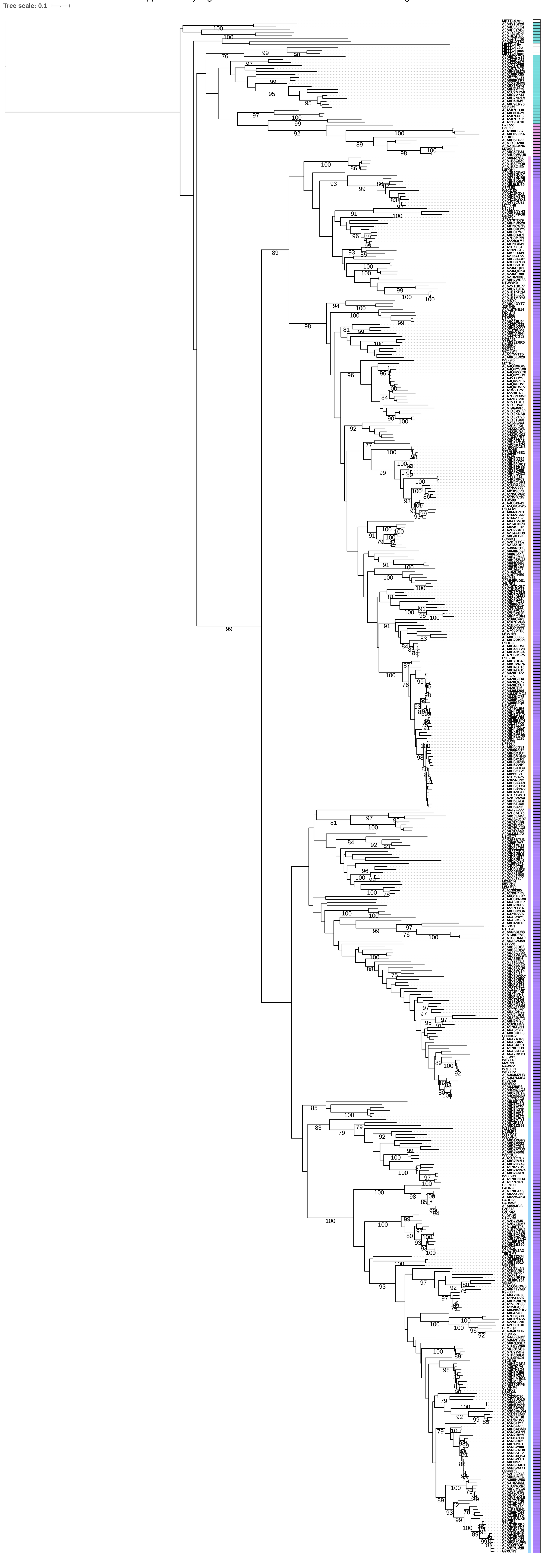

Supplementary Fig. 1D. A maximum likelihood tree of WTAP in fungi

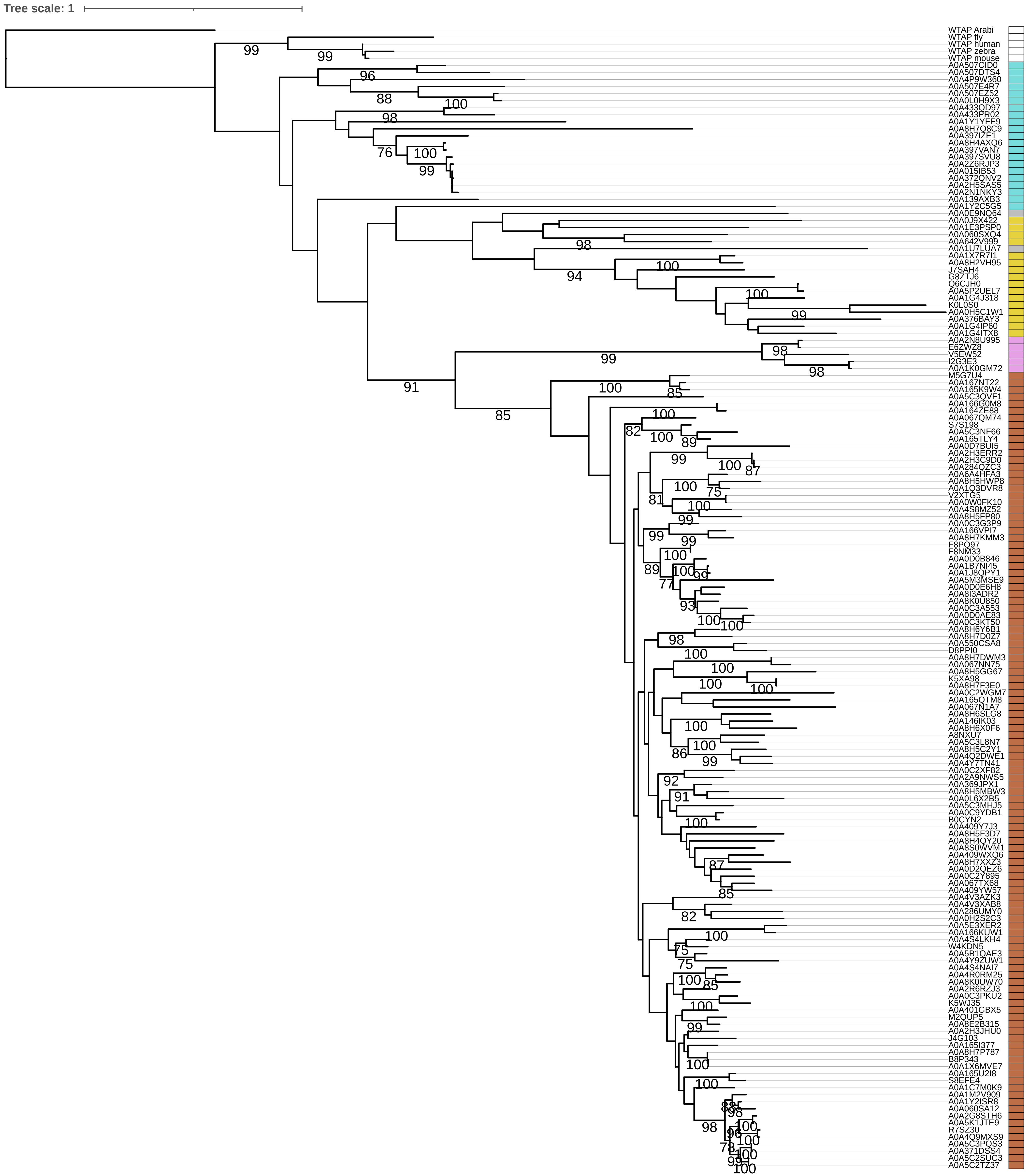

Supplementary Fig. 1E. A maximum likelihood tree of VIRN in fungi

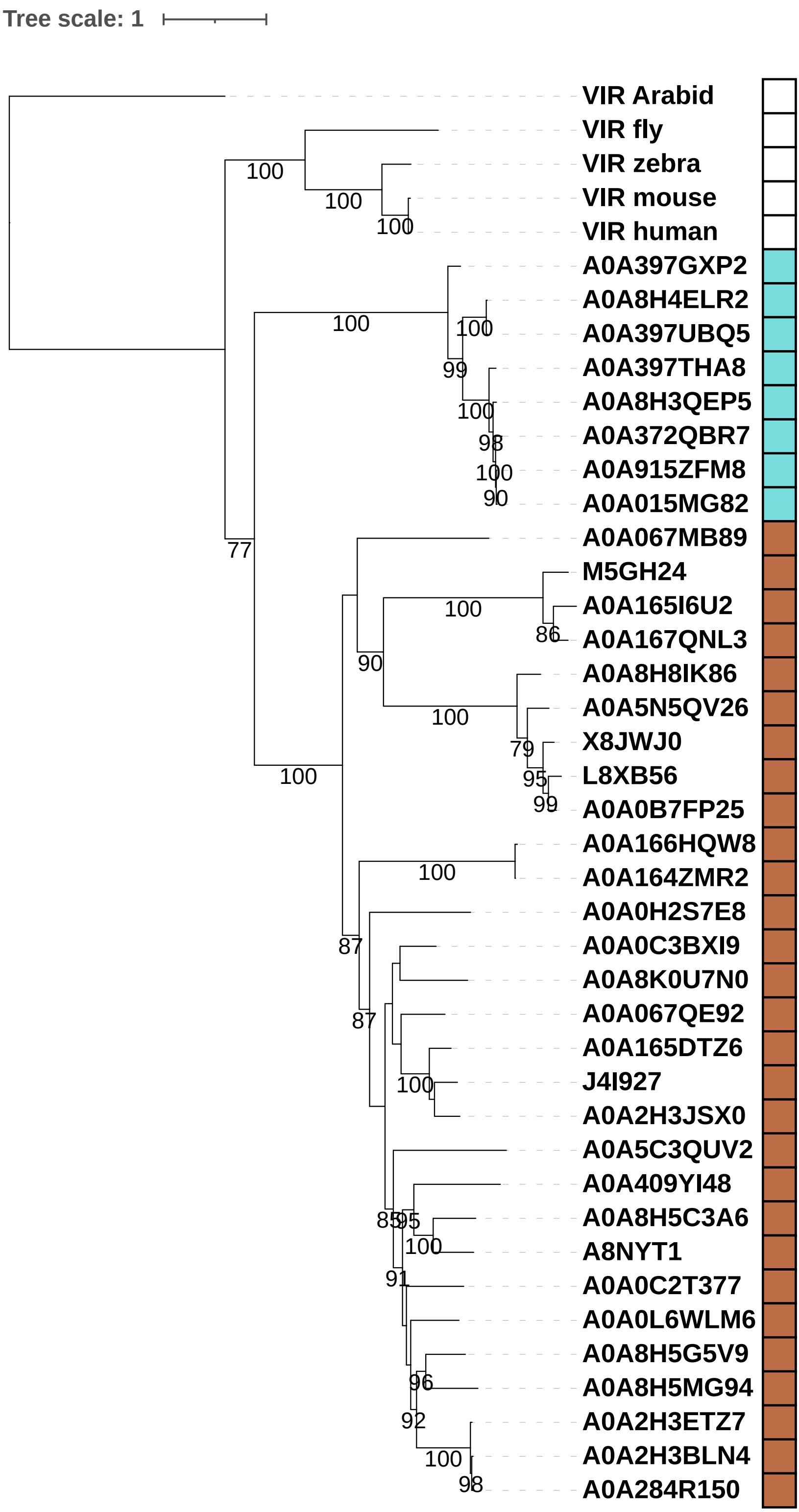

Supplementary Fig. 1F. A maximum likelihood tree of YTH1 in fungi

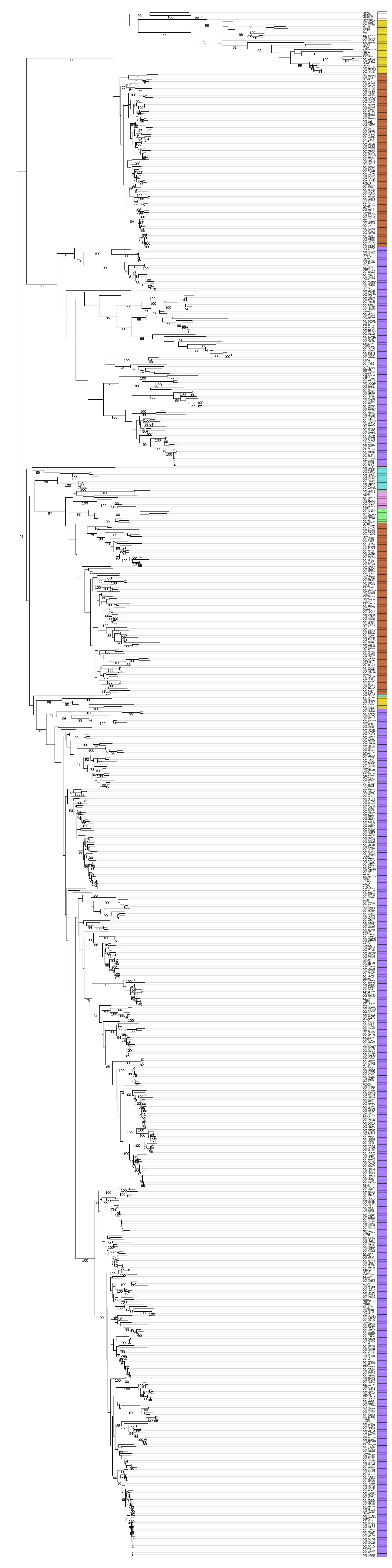

Supplement: Fig. S1 — Maximum likelihood trees of potential m6A factors in fungi. [file msphere.00552-23-s0002.pdf]
