# Supplementary material for: Phylogenetic and functional analyses of N6-methyladenosine RNA methylation factors in the wheat scab fungus Fusarium graminearum
Source: mSphere. 2023 Dec 12;9(1):e00552-23. doi: 10.1128/msphere.00552-23 (PMC10826363; doi:10.1128/msphere.00552-23)
Supplement: Table S2 — Numbers of putative m6A erasers found in 27 selected fungi. [file msphere.00552-23-s0004.docx]

**Supplementary Table S2.** Numbers of putative m^6^A erasers found in 27 selected fungi.

| **Taxa** | **Fungal Class** | **Species name** | **counts** |
| --- | --- | --- | --- |
| Early diverging fungi | Chytridiomycetes | *Spizellomyces punctatus* | 9 |
|  |  | *Batrachochytrium salamandrivorans* | 6 |
|  | Basidiobolomycetes | *Basidiobolus meristosporus* | 10 |
|  | Zoopagomycetes | *Piptocephalis cylindrospora* | 5 |
|  | Mucoromycetes | *Rhizopus microsporus* | 3 |
|  |  | *Mucor circinelloides* | 4 |
| Basidiomycota | Pucciniomycetes | *Melampsora larici-populina* | 4 |
|  | Ustilaginomycetes | *Ustilago maydis* | 3 |
|  | Exobasidiomycetes | *Tilletia caries* | 3 |
|  | Agaricomycetes | *Amanita muscaria* | 4 |
|  | Tremellomycetes | *Cryptococcus neoformans* | 5 |
| Ascomycota | Saccharomycetes | *Yarrowia lipolytica* | 2 |
|  |  | *Wickerhamiella sorbophila* | 2 |
|  |  | *Pichia membranifaciens* | 1 |
|  |  | *Candida auris* | 1 |
|  | Pezizomycetes | *Tuber aestivum* | 5 |
|  |  | *Morchella conica* | 5 |
|  | Leotiomycetes | *Botrytis cinerea* | 5 |
|  | Sordariomycetes | *Fusarium graminearum* | 5 |
|  |  | *Magnaporthe oryzae* | 6 |
|  |  | *Claviceps purpurea* | 5 |
|  |  | *Neurospora crassa* | 5 |
|  | Dothideomycetes | *Alternaria arborescens* | 6 |
|  |  | *Ascochyta lentis* | 6 |
|  | Eurotiomycetes | *Aspergillus oryzae* | 6 |
|  |  | *Aspergillus fumigatus* | 5 |
|  |  | *Monascus purpureus* | 4 |
