# Supplementary material for: Phylogenetic and functional analyses of N6-methyladenosine RNA methylation factors in the wheat scab fungus Fusarium graminearum
Source: mSphere. 2023 Dec 12;9(1):e00552-23. doi: 10.1128/msphere.00552-23 (PMC10826363; doi:10.1128/msphere.00552-23)
Supplement: Table S3 — Primers. [file msphere.00552-23-s0005.docx]

**Supplementary Table S3.** Primers used in this study.

| **Primers** | **Sequence (5'-3')** | **Description/Purpose** |
| --- | --- | --- |
| FGRRES_06225_L5 | CGAACCGAGGATTGCTGGCTTG | Left flanking region |
| FGRRES_06225_L3 | GTTGACCTCCACTAGCTCCAGCCAAGCCAAATCTGACTGCCGGATGACAG |  |
| FGRRES_06225_R5 | GGCAAAGGAATAGAGTAGATGCCGACCGCAGCAACCTGAACACTGGAACAAC | Right flanking region |
| FGRRES_06225_R3 | GCCAGCCATCGAAATTCTTCAC |  |
| FGRRES_06225_N5 | CTCAATCACTTACAGCGCAACCG | Split marker constructs |
| FGRRES_06225_N3 | CCAATTGCCTGGCTTCAAGCTG |  |
| FGRRES_01159_L5 | CGTCTTGTGCTGTTGTAAGTGCC | Left flanking region |
| FGRRES_01159_L3 | GTTGACCTCCACTAGCTCCAGCCAAGCCGATTCCAAGTGGTGACAGGATG |  |
| FGRRES_01159_R5 | GGCAAAGGAATAGAGTAGATGCCGACCGCTTGGTATTGGCATGTTGGAGTG | Right flanking region |
| FGRRES_01159_R3 | CTGTGCCTCTTGAGCTAGTGCAAG |  |
| FGRRES_01159_N5 | GTTGCACTTCGACACTTCACCTC | Split marker constructs |
| FGRRES_01159_N3 | GTACTAGGTATCCGTAGGTGTGC |  |
| FGRRES_16652_L5 | CGACTGGTGATTCTAAGCCTTGC | Left flanking region |
| FGRRES_16652_L3 | GTTGACCTCCACTAGCTCCAGCCAAGCCTCACAGAGATCGACGCTCATGAG |  |
| FGRRES_16652_R5 | GGCAAAGGAATAGAGTAGATGCCGACCGCTGCGTATCTTGAAGACTGGCC | Right flanking region |
| FGRRES_16652_R3 | GCCTCATGCTTAGACAAGAAGTCC |  |
| FGRRES_16652_N5 | GCGATGCCGGTTTCTGAAGTATC | Split marker constructs |
| FGRRES_16652_N3 | CTTGCAGTCGCTAGTTGAAGGAG |  |
| FGRRES_01626_L5 | GTGGTAGTCCAATGACCAGATGG | Left flanking region |
| FGRRES_01626_L3 | CGCTATACATTGATGGTCTGCTGCTGGGAGTTGCTGAAGGATTGCAAG |  |
| FGRRES_01626_R5 | GGAGACGAGATCAAGCAGATCAACGGCGAATGACATATTGGAAGCGG | Right flanking region |
| FGRRES_01626_R3 | CTGCTGAAGTTCTGGAGTTGGC |  |
| FGRRES_01626_N5 | CACCCACGCTTACATAGAACCG | Split marker constructs |
| FGRRES_01626_N3 | GTTGGCAGGCGTAAGTTCATTCC |  |
| FGRRES_06249_L5 | GGTCATCGGTTACTGGAATTCGTC | Left flanking region |
| FGRRES_06249_L3 | GTTGACCTCCACTAGCTCCAGCCAAGCGACGATGCTGAAGAACAGCATAGC |  |
| FGRRES_06249_R5 | GGCAAAGGAATAGAGTAGATGCCGACCGGGAGGAATGCTACGGAATGTACC | Right flanking region |
| FGRRES_06249_R3 | CATCCTCTGCACATCCTCTGATC |  |
| FGRRES_06249_N5 | CTTGTTGGAACTCACACGTCGC | Split marker constructs |
| FGRRES_06249_N3 | GCTTCGAGTGGAATCTGCTTCTC |  |
| HYG-F | GCTTGGCTGGAGCTAGTGGAG | Split marker constructs |
| HYG-R | CGGTCGGCATCTACTCTATTCCTT |  |
| YG-F | CGATGTAGGAGGGCGTGGATATGTC | Split marker constructs |
| HY-R | TGTAGTGTATTGACCGATTCCTTGCG |  |
| HYG_check_fwd | GCTTGGCTGGAGCTAGTGGAG | PCR check |
| HYG_check_rev | CGGTCGGCATCTACTCTATTCCTT |  |
| MTA1_RT_fwd | CCTCTACATGGTTCTATTGAGGCC | RT-PCR |
| MTA1_RT_rev | GATTCCGAAGATTCGGCTTTCG |  |
| MTA1_RT_rev2 | CATCTCTGTCAAGTTGGAGACG | realtime RT-PCR |

**Supplementary Table S3** (continued)

| **Primers** | **Sequence (5'-3')** | **Description** |
| --- | --- | --- |
| EF1a_realT_fwd | GGCTTTCACCGACTACCCTC | realtime RT-PCR |
| EF1a_realT_rev | CTTCTCGACGGCCTTGATG |  |
| EF1a_RT_fwd | GTATCGACAAGCGAACCATC | RT-PCR; expected size  (gDNA 658 bp, mRNA: 356 bp) |
| EF1a_RT_rev | GGCAACAATGAGGTTCTTGAC |  |
| MTA1_OE_fwd | CTAACAGCTACAGATCATGACTAAAGTCAGCCATCGG | Infusion cloning to pDS23 |
| MTA1_OE_rev | CTTGCTCACCATAAGCTTTTCTATGTTGTTCCAG |  |
| MTA1_OE_RT_fwd | CAGCTACAGATCATGACTAAAGTCAGCC | RT-PCR; expected size with MTA1_RT_rev, 880 bp |
